# Electrolyte Transport Parameters and Interfacial Effects in Calcium Metal Batteries: Analogies and Differences to Magnesium and Lithium Counterparts

**DOI:** 10.1002/advs.202506498

**Published:** 2025-06-25

**Authors:** Joachim Häcker, Tobias Rommel, Laurin Rademacher, Sibylle Riedel, Zhirong Zhao‐Karger, J. Alberto Blázquez, K. Andreas Friedrich, Maryam Nojabaee

**Affiliations:** ^1^ Institute of Engineering Thermodynamics German Aerospace Center (DLR) Pfaffenwaldring 38‐40 70569 Stuttgart Germany; ^2^ Helmholtz Institute Ulm (HIU) Electrochemical Energy Storage Helmholtzstrasse 11 89081 Ulm Germany; ^3^ Institute of Nanotechnology (INT) Karlsruhe Institute of Technology (KIT) Hermann‐von‐Helmholtz Platz 1 D‐76344 Eggenstein‐Leopoldshafen Germany; ^4^ CIDETEC Basque Research and Technology Alliance (BRTA) P° Miramon, 196 Donostia‐San Sebastian 20014 Spain; ^5^ Institute of Building Energetics, Thermotechnology and Energy Storage (IGTE) University of Stuttgart Pfaffenwaldring 6 70569 Stuttgart Germany

**Keywords:** EIS, electrolyte transport, metal anode battery, multivalent metals, separator tortuosity

## Abstract

Magnesium and calcium metal batteries are promising emerging technologies. Their high capacity and low redox potential translate to a high theoretical energy density, making them attractive candidates for future energy storage solutions. Owing to their neighboring position and the diagonal relationship in the periodic table to lithium, Mg^2+^, Ca^2+^, and Li^+^ ions feature commonalities in terms of ionic radius, carried charge, and charge density. The present study aims to shed light on the similarities but also differences of Ca electrolytes and metal anodes in comparison to their Mg and Li counterparts in terms of transport properties and processes at the anode/electrolyte interface, respectively. To ensure comparability, an electrolyte comprising B(hfip)_4_^−^ anions in monoglyme is applied in either case. By executing galvanostatic polarization and pulsing with different separator materials, the separator tortuosity, diffusion coefficient, and transference number are determined. Further, the charge transfer characteristics as well as the adsorption layer and solid electrolyte interphase formation are investigated by electrochemical impedance spectroscopy. The cation charge density was found to be crucial for diffusion and desolvation processes, yet surprisingly, also a cation‐dependent separator tortuosity was observed. The study concludes with a recommendation on suitable separators for each metal battery system.

## Graphical abstract

Mg and Ca metal batteries represent promising alternatives to Li‐based batteries. Due to their position in the periodic table, the metals and their corresponding cations share commonalities, which lead to similarities but also differences in terms of electrolyte transport properties and interfacial processes. These are investigated herein by means of polarization and EIS to reveal charge transfer characteristics and identify suitable separators.

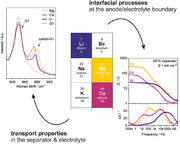

## 1 Introduction

The use of lithium metal as anode material is considered the holy grail in rechargeable battery research due to the lowest overall redox potential (−3.04 V vs SHE) and the high gravimetric and volumetric capacity of 3860 mAh g^−1^ and 2060 mAh cm^−3^, respectively. However, its strong tendency to dendrite formation and oxidation hinders its application due to safety concerns and the lack of cycling stability. Furthermore, lithium exhibits a limited abundance in the Earth's crust (0.0020%) bearing the risk of high pricing and shortages. Therefore, other metals like Na, K, Mg, Ca, Zn, or Al are researched as battery anode in recent years. While they are all providing sufficient abundance (1.5–8.1% in the Earth's crust), earth alkaline metals (Mg, Ca) and post‐transition metals (Al, Zn) are generally regarded safer than alkaline metals (Na, K) as they are less prone to dendritic metal deposition. Among the multivalent candidates, Ca combines the lowest redox potential (−2.87 V vs SHE) with a high gravimetric and volumetric capacity of 1330 mAh g^−1^ and 2070 mAh cm^−3^, respectively. Hence, the neighbored position in the periodic table, Ca^2+^ exhibits similarities to Mg^2+^ and Li^+^ ions in terms of carried charge (2e) and charge density (76 vs 87 C mm^−3^), respectively, while featuring a larger ionic radius (100 vs 72 vs 76 pm).^[^ ^1^ ^]^ The ion specifications largely affect the electrolyte transport properties, which was nicely illustrated by Mandai et al. in their recent comprehensive comparison of mono‐ and divalent cation electrolytes comprising weakly coordinating B(hfip)_4_^−^ anions in monoglyme (G1).^[^ ^2^ ^]^ The here presented study is complementary as it examines the relationship between transport properties in the electrolyte and the nature of different wetted separator materials with interfacial processes at the metal anode.

Although being an electrochemically inactive material, the separator affects the battery performance in significant manner and has to fulfill several requirements. Above all, it has to be electrically insulating while allowing fast ion diffusion. Further, it should provide sufficient chemical, electrochemical, thermal, and mechanical stability and a good wettability by the electrolyte. Finally, a uniform separator porosity is desired, as otherwise uneven current density distributions and consequently inhomogeneous metal stripping and plating at the anode may result, leading to undesired changes in anode morphology and even dramatic dendrite formation to cause relevant safety concerns. Due to its mechanical strength and chemical stability, the research on LIB and LMB commonly applies thin polyolefin materials like Celgard 2500 (polypropylene, PP, 25 µm, 64 nm pores, 55 % porosity). In contrast, the studies on Mg and Ca metal batteries mostly utilize Whatman separators (glass fiber, > 200 µm, > 1 µm pores, > 90 % porosity) due to its superior cycling stability. Indeed, glass fibers are superior to PP in terms of voltage and thermal stability and as well as electrolyte wettability,^[^ ^3^ ^]^ yet, its usually 10 times higher thickness and large electrolyte uptake making them no practical choice to achieve high‐energy density batteries. While a lot of studies are conducted to improve the Mg metal cell performance by separator coatings (SuperP,^[^ ^4^ ^]^ CNF,^[^ ^5^ ^]^ rGO,^[^ ^6^ ^]^ TiS_2_,^[^ ^7^ ^]^ Mo_6_S_8_,^[^ ^8^ ^]^ or POM clusters^[^ ^9^ ^]^), only few comprise thin separators like Entek ET 20–60 (PE, 20 µm, 37% porosity),^[^ ^10^, ^11^ ^]^ polyimide (PI),^[^ ^12^ ^]^ Celgard 2400 (PP, 25 µm, 41% porosity),^[^ ^13^ ^]^ or Celgard 2500^[^ ^14^ ^]^—with their cell functioning either relying on a Li^+^/Mg^2+^ hybrid electrolyte, relative elevated temperatures up to 80°C, Cu redox mediators, low C‐rates (0.01C) or excess electrolyte volume, respectively.

In that context, the separator tortuosity is a crucial property, which is determined herein in addition to the binary diffusion coefficient and cation transference number for the specific (Mg, Ca, Li) electrolyte system—in either case applying the weakly coordinating hexafluoroisopropyloxy borate B(hfip)_4_^−^ (hfip = OCH(CF_3_)_2_) in monoglyme (G1), which is regarded the current benchmark electrolyte in the Mg and Ca metal battery research.^[^ ^15^, ^16^ ^]^ Further, the separator selection—and the corresponding differing electrolyte volume—influences the interfacial processes at the anode surface due to different physical properties (e.g. porosity, pore size) and the chemical nature of the separator (e.g. polarity). Therefore, electrochemical impedance spectroscopy (EIS) is applied to provide insights into the charge transfer process and potentially forming surface layers such as the solid electrolyte interphase (SEI) and the adsorption layer. The formation of latter is surveyed during OCV with the Ca system behaving similar to the Mg system—at least with large electrolyte volume and thick separator. While in the Mg system, the processes at the anode/electrolyte interface, namely the desolvation and ongoing SEI formation, are dominant, the bulk electrolyte properties are most relevant in the Ca system. The Li system generally features low overpotentials with the resilience of the separator toward lithium dendrites being the most crucial property.

Overall, it is aimed to give valuable insights into the metal and cation characteristics, identify suitable separator materials and especially accelerate the development of Ca metal batteries by transferring gained knowledge and promising approaches from the research on Mg and Li metal batteries.

## 2 Results and Discussion

### 2.1 Metal and Cation Characteristics

The characteristics of Mg, Ca, and Li metal along with their corresponding ions and electrolytes are summarized in **Table** **1**. The electrochemical properties as well as element abundance and costs are already pointed out above with the realization of a Li metal anode being the ultimate goal, while Ca and Mg offering cost (abundancy in the earth crust: 2,3 % Mg; 4,2 % Ca; < 0,01% Li), safety (less tendency for dendrite formation) and recycling advantages (current Mg recycling rate of 25–50 % vs < 1 % for Li). Regarding the manufacturing of thin free‐standing foils (20–50 µm), Li metal most often requires a copper substrate to maintain R2R processability, which adds costs and weight. In contrast, Ca and Mg metal are superior to Li metal in terms of larger stiffness, but are rather hard (Ca) or lack of sufficient ductility (Mg). Latter is circumvented by grain refinement (< 1 µm) and activation of slip systems (non‐basal) to actually realize thin Mg foils (< 45 µm).^[^ ^17^, ^18^ ^]^

**Table 1:** Properties of Mg, Ca, and Li metal and their corresponding ions and electrolytes.^[^ ^19^, ^20^ ^]^

	Mg	Ca	Li
E^0^ vs. SHE / V	−2.37	−2.87	−3.04
Specific capacity / mAh g^−1^	2205	1330	3861
Volumetric capacity / mAh cm^−3^	3833	2070	2066
Density / g cm^−3^	1.738	1.550	0.534
Atomic mass / g mol^−1^	24.31	40.08	6.94
Crystal structure	hcp	fcc	bcc
Stiffness (Young's modulus E) / GPa	45	20	4.9
Elastic shear stiffness (Shear modulus G) / GPa	17	7.4	4.2
Brinell hardness / MPa	44–260	170–416	5
Melting point / °C	650	842	180.5
Abundance in Earth's crust (x^th^ element) / ppm	23.000 (7th)	42.000 (5th)	20 (32th)
Commodity quotes / USD t^−1^	300–1000	200–500	7000–8000
Carried charge / e	2	2	1
Shannon effective ionic radius (CN)^[^ ^1^ ^]^ / pm	72 (6)	100 (6)	76 (6) 59 (4)
Stokes radius in water^[^ ^21^ ^]^ / pm	347	310	238
Charge density (CN) / C mm^−3^	205 (6)	76 (6)	87 (6) 186 (4)
Polarization strength / C m^−2^	14.7	10.4	8.1
Unit cell volume M[B(hfip)_4_]_n_ * x G1 / Å	na	14098.5 (at 180 K)^[^ ^22^ ^]^	na
Conductivity 0.2 M M[B(hfip)_4_]_n_ in G1 / mS cm^−1^	9.02	8.25	6.08

Due to Mg^2+^ ions exhibiting a similar ionic radius to Li^+^ while carrying a doubled charge, a high charge density of 205 C mm^−3^ results. As Ca^2+^ ions feature a larger radius, the charge density is significantly smaller (76 C mm^−3^) and even lower than Li^+^ (87 C mm^−3^). The “softness” of Ca^2+^ and Li^+^ evokes lower energy barriers for both the solid‐state diffusion and the desolvation consequently enhancing the electrode kinetics and interfacial processes, respectively.^[^ ^23^, ^24^ ^]^ For example, lower formation energies for [Li(G1)]^+^ (−61.0 kcal mol^−1^)^[^ ^25^ ^]^ and [Ca(G1)]^2+^ (−111.9 kcal mol^−1^)^[^ ^26^ ^]^ are found compared to [Mg(G1)]^2+^ (−157.6 kcal mol^−1^)^[^ ^26^ ^]^ complexes.

**Figure 1:**
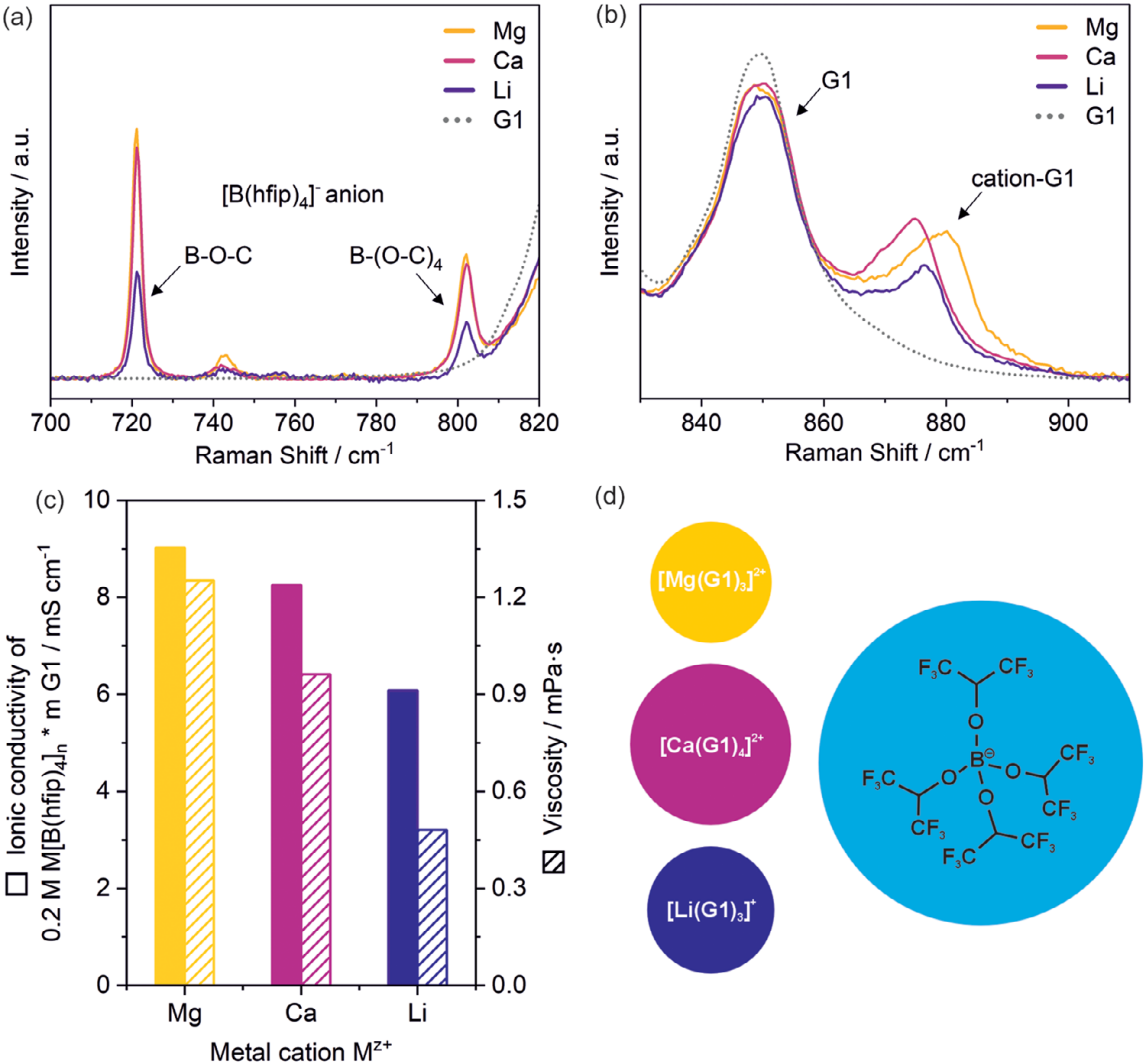
a,b) Raman spectra of the 0.2 m Mg[B(hfip)_4_]_2_/G1, 0.2 m Ca[B(hfip)_4_]_2_/G1 and 0.2 m Li[B(hfip)_4_]/G1 electrolytes. c) Ionic conductivity and viscosity as well as d) the molecule structure and number of coordinating G1 molecules in M[B(hfip)_4_]_n_ / G1 electrolytes and salts, respectively.

In general, softer ions have weaker interactions with their surrounding molecules in solution. Whether the cation‐solvent or the cation‐anion (e.g. ion pairing) interaction is stronger, the tendency for solvent or anion decomposition along cation reduction (plating process) is larger, respectively.^[^ ^23^, ^27^ ^]^ This greatly depends on the solvent and anion choice and has to be further distinguished for monovalent and divalent cations forming contact ion pairs (CIP) and solvent‐separated ion pairs (SSIP).^[^ ^2^ ^]^ To gain distinct insights into the solvation shell and ion pairing, Raman spectroscopy was performed (Figure S4, Supporting Information). Following the interpretation for the Li[B(hfip)_4_] ∙ 3 G1 salt by Roy et al.^[^ ^28^ ^]^ the band at 720–725 cm^−1^ and 800–805 cm^−1^ is assigned to the B‐O‐C and B‐(O‐C)_4_ breathing mode of the [B(hfip)_4_]^−^, respectively (**Figure** **1**a). Latter was also observed in Ca[B(hfip)_4_]_2_ / G1 electrolytes as sharp feature at 801 cm^−1^.^[^ ^29^ ^]^ Backed by the findings of Tchitchekova et al.^[^ ^30^ ^]^ the band at 870–885 cm^−1^ is assigned to the solvent‐cation interaction, shifted by approx. 20–30 cm^−1^ from the main G1 band at 850 cm^−1^ (Figure 1b). The shift increases with increasing charge density (Ca^2+^ < Li^+^ < Mg^2+^) indicating the strong Mg^2+^‐G1 interaction. According to Hahn et al., the shoulder at 865–875 cm^−1^ can be assigned to a feature of the [B(hfip)_4_]^−^ salt.^[^ ^29^ ^]^

The findings are in line with recently reported ion pair ratios for MgTFSI/G1 and Mg[B(hfip)_4_]_2_/G1 solutions of < 20 %^[^ ^31^ ^]^ and Mg^2+^: Mg(WCA)^+^ 4:1,^[^ ^32^ ^]^ respectively. In comparison, a cation ratio of Ca^2+ ^: Ca(WCA)^+^ 3:2 is reported for Ca[B(hfip)_4_]_2_/G1, confirming a stronger bound solvation shell for Mg^2+^ shielding its high charge density toward the B(hfip)_4_^−^ anion hindering the formation of ion pairs. Thus, the dissociation degree is higher compared to Ca^2+^ and Li^+^ and consequently the concentration of free charge carriers of the 0.2 M Mg[B(hfip)_4_]_2_ / G1 electrolyte is higher. This results in higher ionic conductivities, while the slightly higher viscosity points to a lower ion mobility (Figure 1c).

The significantly lower conductivity of the 0.2 M Li[B(hfip)_4_] electrolyte despite a favorable lower viscosity compared to its Ca counterpart with similar cation charge density is mainly originated in the lower net charge concentration (anions + cations). However, also with the same net charge concentration in a 0.4 m Li[B(hfip)_4_] / G1 electrolyte a slightly lower conductivity and viscosity is observed (Figure S5, Supporting Information) confirming previous results.^[^ ^2^ ^]^ This is assigned to i) a smaller ionic radius of Li^+^ (100 pm vs 76 pm) inducing less steric hindrance for ion pair formation and consequently a lower dissociation degree and ii) Li^+^ being reported to form a tetracoordinated complex of bidental coordinating G1 and a [B(hfip)_4_]^−^ anion in contrast to the divalent cations, which are completely stabilized by the G1 molecules to provide isolated charge carriers.^[^ ^2^ ^]^ However, in that context only one coordinating G1 is identified,^[^ ^2^ ^]^ while in agreement with Roy et al.,^[^ ^28^ ^]^ the NMR spectra of the dried Li[B(hfip)_4_] salt herein indicate the incorporation of three G1 molecules (Figure S3, Supporting Information), on which basis the molecular weight is determined. With two coordinating site per G1 molecule a coordination number of 6 results for Li^+^ herein, while literature reports preferential coordination numbers of CN = 4–5 for Li^+^.^[^ ^2^, ^15^, ^16^, ^30^, ^31^, ^33^, ^34^ ^]^ The reported CN = 6–7 for Mg^2+^ and CN = 6–8 for Ca^2+^ are in line with the three and four G1 molecules, respectively, determined herein.

Overall, Figure 1 illustrates the highly dissociating nature of the M[B(hfip)_4_]_n_ salt to provide high conductivities (i.e. a large number of free charge carriers) even at low molarities—remarkable in the non‐polar solvent G1 for which the ionicity was found to be rather low compared to other solvent, indicating a lower dissociation capability and higher ratio of ion pairing.^[^ ^33^ ^]^

### 2.2 Separator Tortuosity

**Figure 2:**
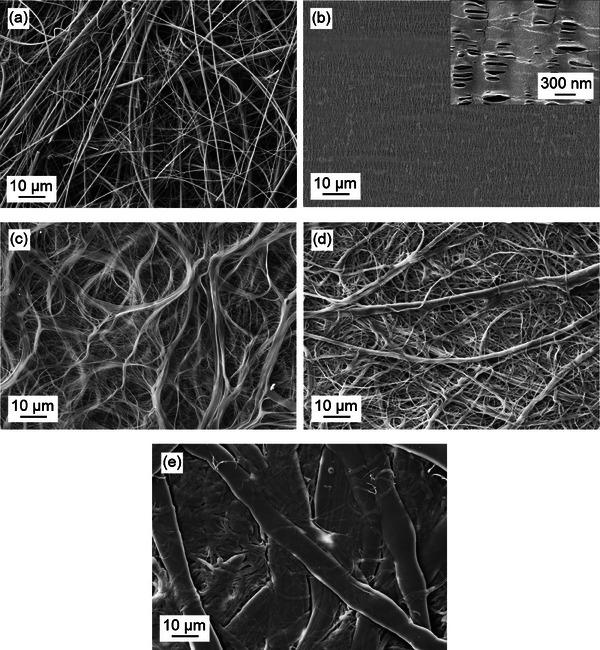
Separator morphology of a) Whatman GF/C (glass fiber), b) Celgard 2500 (PP), c) Solupor 3P07A (PE), d) NKK TBL4620 (cellulose), and e) Dreamweaver Silver 25 (cellulose).

To study the influence on the cell performance, five commercial separator materials are selected (**Table** **2**). Glass fiber separators like Whatman GF/C are commonly used to investigate new chemistries as it does not mitigate the ion flux and is insensitive to early short‐circuits due to the large pores/porosity (**Figure** **2**a) and thickness, respectively. It further exhibits excellent wettability due to the polar surfaces and is capable to soak a large electrolyte volume. Thus, it serves as benchmark material for lab cells in this study, yet has apparently no practical relevance due to its thickness. In contrast, Celgard 2500 (Figure 2b) represents the benchmark material for practical cells as it is the most common separator material in LIB. It is composed of PP and exhibits small pores (64 nm) and porosity (55 %), which is reflected in a high Gurley number (200 s), i.e. the air permeability, which is proportional to the electrical resistance of the separator material.^[^ ^35^ ^]^

**Table 2:** Separator properties.

Separator	Chemical composition	Thickness d / µm	Pore size / µm	Porosity ε / %	Gurley / s	Wettability with ethers
Whatman GF/C	Oxides	260	1.2	93*	6.7	+
Celgard 2500	PP	25	0.064	55	200	‐
Lydall Solupor 3P07A	PE	28	0.7	83	1.4	o
NKK TBL4620	Cellulose	16	na	69	na	+
Dreamweaver Silver 25	Cellulose	27	1.1	56	80	+

*determined by Singh et al.^[^ ^3^ ^]^

Lydall Solupor 3P07A (Figure 2c) is another polyolefine‐based separator (PE), yet with significantly varying properties to Celgard 2500. Among the common thin separators, Solupor 3P07A comes closest to the Whatman GF/C properties also exhibiting a large pore size (0.7 µm) and porosity (83 %), however, the non‐polar PE structure shows worse wetting behavior.

In contrast, the cellulose‐based paper‐like NKK TBL4620 (Figure 2d) and Dreamweaver Silver 25 separator (Figure 2e) provide excellent wettability—however only moderate porosity. Dreamweaver comprises micro‐ and nanofibers to combine mechanical strength with small pores and a narrow pore size distribution, respectively.

Among the separator properties, the tortuosity is of great interest as it describes the average pore conductivity and ionic transport applying the actual electrolyte solution instead of a gas flux in case of the Gurley number. As it further considers the thickness of the separator, a direct comparison of materials is enabled. The tortuosity τ equals 1 for parallel cylindrical pores and >1 for a hindered ion diffusion—it can be calculated via

(1) $$\tau =\frac{R_{s}\cdot \sigma \cdot \varepsilon \cdot A}{d}$$

with *ε*, *A* and *d* being the porosity, area and thickness of the separator, respectively (Table 2). The ionic conductivity of the bulk electrolyte solution σ and the resistance of the wetted separator *R_s_* are determined via a conductivity electrode and electrochemical impedance measurements in Cu|Cu cells with subsequent fitting of the gained spectra (Figure S6, Supporting Information), respectively. For latter, an equivalent circuit model (ECM) for blocking electrodes is applied for most spectra due to the missing charge transfer at the blocking Cu surface. For some spectra an R‐CPE element is used in addition to achieve a more accurate fitting. The tortuosity *τ*, calculated from the derived *R_s_* values, are listed in Table S1, Supporting Information. Therein, also the MacMullin number *N_M_* is given, which is defined by the ratio of the resistivity, conductivity or diffusion coefficient of the wetted separator (*R_s_, σ_s_, D_s_*) and the bulk electrolyte solution (*R_e_, σ_e_, D_e_*).^[^ ^36^ ^]^

(2) $$N_{M}=\frac{R_{s}}{R_{e}}=\frac{\sigma_{e}}{\sigma_{s}}=\frac{D_{e}}{D_{s}}=\frac{\tau }{\varepsilon }$$

Typical McMullin numbers are in the range of 4–10 with smaller numbers being desired as it reflects a lower separator resistance.

**Figure 3:**
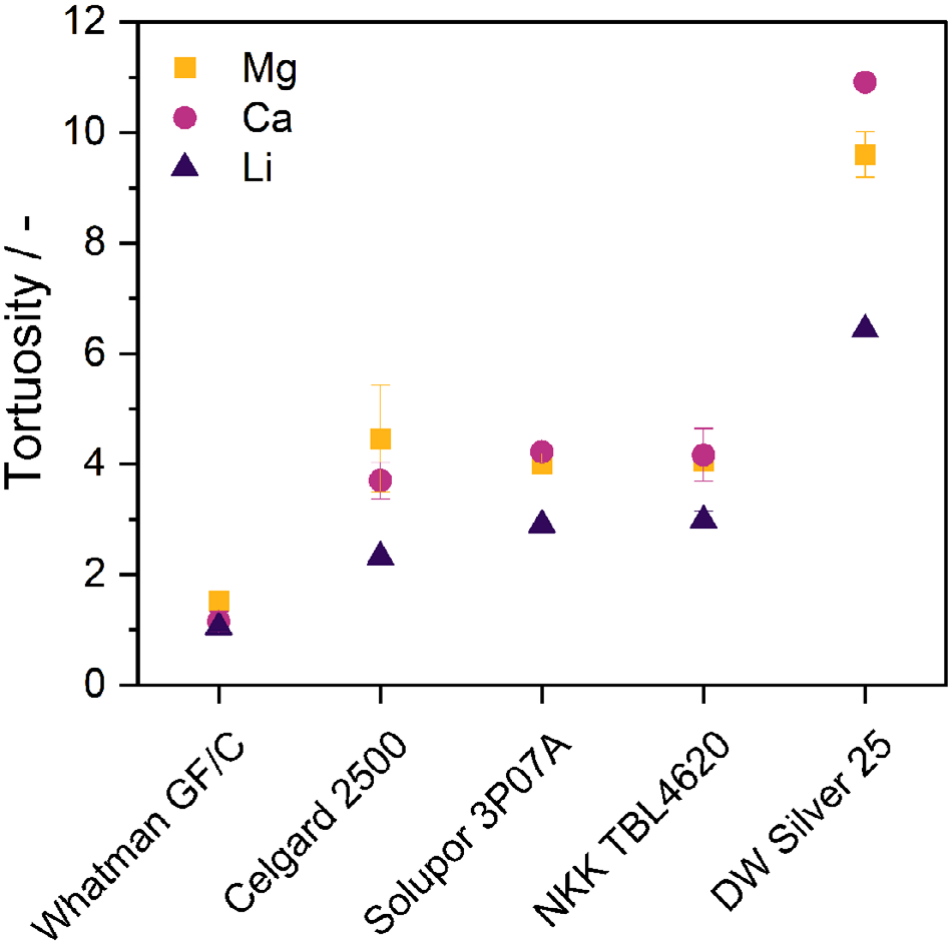
Calculated separator tortuosity in dependence on the cation system.

The tortuosity values and the influence of the different cation systems are visualized in **Figure** **3**. GF/C exhibits the lowest tortuosity (τ = 1.1–1.5) due to its open porosity, which is beneficial for the ionic transport, but prone to penetration by metal deposits/dendrites. Celgard, Solupor and NKK exhibit medium values (τ = 2.3–4.5), while the tortuosity of the Dreamweaver reflects a highly tortuous fiber network (τ > 6).

More interestingly, the tortuosity values indicate a dependence on the cation in the electrolyte—with increasing influence at higher tortuosities. This is in fact surprising as the tortuosity is regarded a separator‐specific property, with Landesfeind et al. confirming its independence on the electrolyte salt, solvent and concentration—however only comparing different anions in lithium electrolytes.^[^ ^37^ ^]^ Since the cation‐dependent phenomenon herein is at least qualitatively reproducible and has been observed within different separators, its potential origin is further elaborated. The apparent tortuosity is influenced by the separator wettability, electrolyte viscosity, as well as the cation charge density and solvation shell size. Considering the different cations in the present study, the altered tortuosity results from the cation characteristics pointed out above—despite the same electrolyte concentration, anion, and solvent. While the differences in wettability and viscosity of the different electrolytes are rated negligible, again, the ionic radius and charge density of the cations are regarded decisive: Exhibiting the largest ionic radius and highest G1 coordination number, Ca^2+^ carries the largest solvation shell and experiences more steric hindrance than Mg^2+^ and Li^+^ ions—especially in networks with narrow pores. Indeed, the materials herein exhibit larger pores, yet the ion mobility in the convoluted paths is most likely impeded. On the other hand, a higher ion charge density can cause ions to interact with the separator surface. In fact, studies on gas diffusion in mesoporous silica point out that the adsorption at the surface of the porous network varies for different species, temperatures, and pressures—and consequently, the tortuosity differs.^[^ ^38^ ^]^ Therein, the pore size distribution and network topology is regarded crucial—with the apparent tortuosity especially varying with the applied gas species in case of narrower pores, i.e. larger pore wall interactions (inapplicable Knudsen diffusion model).^[^ ^38^ ^]^ Apart from the separator and electrolyte properties, the adsorption layer formation in Mg and Ca electrolytes and potentially altered interfacial chemistry at the electrodes (see below) might influence the values to a certain extent.

### 2.3 Diffusion Coefficient and Cation Transference Number

To further quantify the transport properties, the effective binary diffusion coefficient *D_±_* and the cation transference number *t_+_* are determined. For deriving *D_±_*, the Harned and French method for dilute solution^[^ ^39^ ^]^ (verified by Newman and Chapman for concentrated solutions^[^ ^40^ ^]^) was adopted which relies on inducing a small concentration gradient in the electrolyte by applying a low DC pulse polarization current *I_p_* (*U_p_* < 10 mV). Therein, the Nernst–Einstein equation has to be fulfilled, i.e. no ion association (ion pair formation), which is at least more likely in the low‐concentrated electrolytes used in this study (0.2 m)—neglecting potentially mobile ion pairs. By evaluating the voltage relaxation after pulsing (*I* = 0), the binary diffusion coefficient D_±_ is derived via

(3) $$D_{\pm ,eff}=\frac{l^{2}}{\pi^{2}\cdot t^{\delta }}\cdot \tau =\frac{l^{2}}{\pi^{2}}\cdot m_{ln}\cdot \tau$$

**Figure 4:**
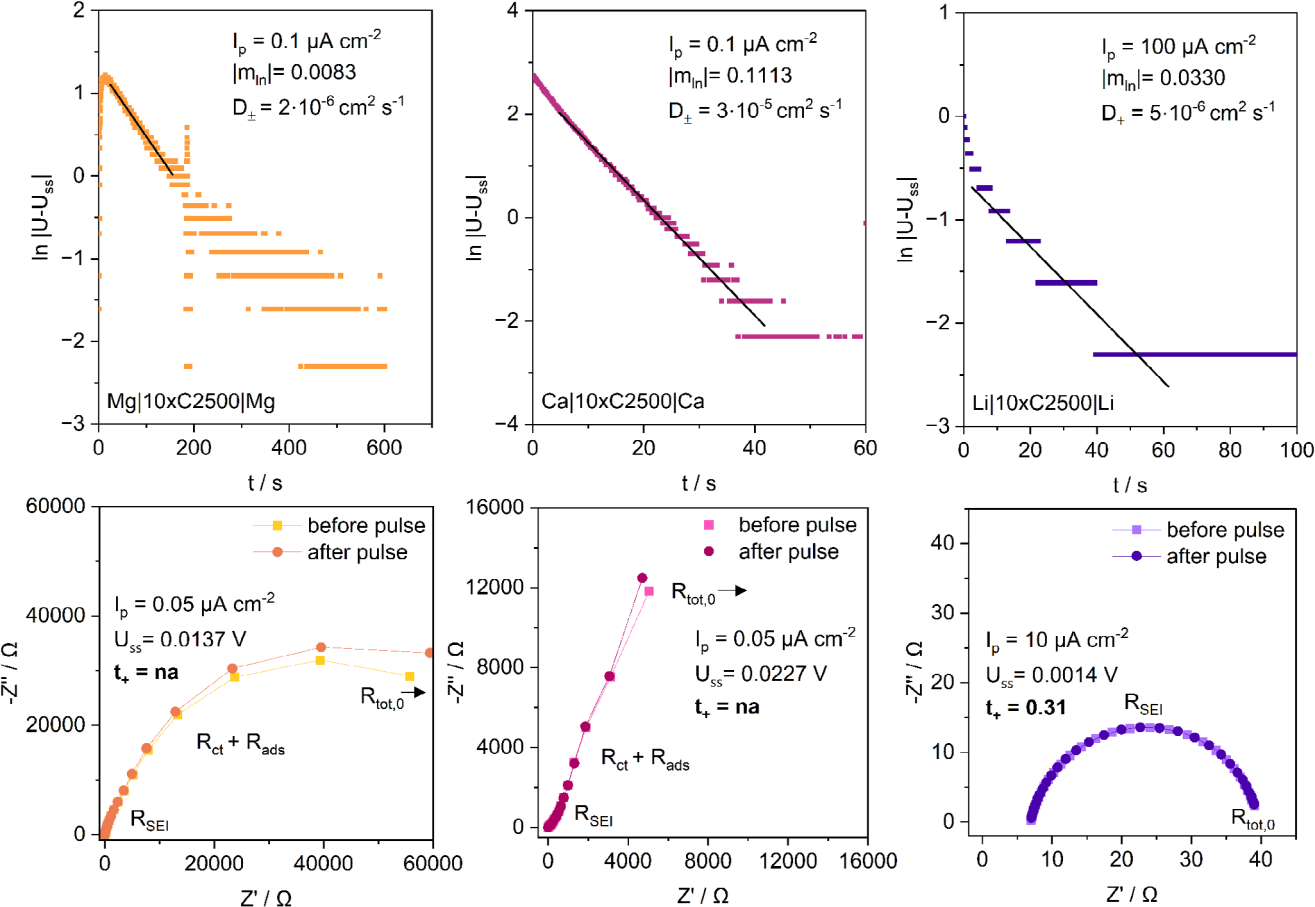
Determination of the binary diffusion coefficient *D_±_* (top) and transference number *t_+_* (bottom) from DC pulse polarization of symmetrical cells with Celgard 2500 separator (10 layers).

With *l, τ *and *t* ^δ^ being the diffusion length (separator thickness), the separator tortuosity and the time constant of the stoichiometry relaxation, respectively.^[^ ^41^, ^42^ ^]^ Latter is determined from the slope |*m_ln_* | of the ln |U(t)‐U_ss_| vs. *t* plot (**Figure** **4**, top).

The 0.2 m Mg[B(hfip)_4_]_2_ electrolyte exhibits the lowest diffusion coefficient of 2∙10^−6^ cm^2^ s^−1^, which is similar to 0.3 m APC, especially with added LiCl,^[^ ^43^ ^]^ but one order of magnitude higher than most other Mg electrolytes like 0.5 m Mg(HMDS)Br,^[^ ^43^ ^]^ Mg(HMDS)_2_/MgCl_2_,^[^ ^44^ ^]^ or 1.25 m MgTFSI (for Mg^2+^).^[^ ^45^ ^]^ Thus, the diffusion coefficient is almost comparable to the 0.2 m Li[B(hfip)_4_] electrolyte (D_±_ = 5∙10^−6^ cm^2^ s^−1^)‐cf. 1.31∙10^−6^ cm^2^ s^−1^ for [Li(G1)_3_][TFSI],^[^ ^46^ ^]^ 2∙10^−6^ cm^2^ s^−1^ for 0.5 m LiClO_4_ in EC:DEC,^[^ ^41^ ^]^ and 5∙10^−6^ cm^2^ s^−1^ for 0.5 m LiPF_6_ in EC:DEC^[^ ^47^ ^]^. Remarkably, the 0.2 m Ca[B(hfip)_4_]_2_ electrolyte shows an even higher value of 3∙10^−5^ cm^2^ s^−1^, which is backed by Hahn et al. reporting a partial diffusion coefficient for the Bhfip^−^ anion of 6∙10^−5^ cm^2^ s^−1^.^[^ ^48^ ^]^ With the [Ca(G1)_4_]^2+^ complex being assumed to exhibit restricted diffusion due to its size, the anion has to be the predominant charge carrier.

To confirm this assumption, the cation transference number *t_+_* (0 ≤ *t_+_* ≤ 1) is determined applying the Bruce–Vincent method via

(4) $$t_{+}=\frac{U_{+}}{U_{tot}}=\frac{I_{p}R_{tot,0}-I_{p}R_{SEI,0}}{U_{ss}-I_{p}R_{SEI,ss}}$$

wherein *U_+_* divided by *U_tot_* represents the voltage fraction of the cation to the total number, respectively. *I* and *U_ss_* are the applied current and steady pulse polarization voltage, and *R_tot,0_*, *R_SEI,0_*, and *R_SEI,ss_* are estimated from EIS spectra gained prior and after pulsing (Figure 4, bottom). Unfortunately, for Mg[B(hfip)_4_]_2_ and Ca[B(hfip)_4_]_2_ no reliable values could be derived due to an adsorption layer with large impedance forming in non‐current conditions (see subsequent chapters), resulting in large errors. At least for Li[B(hfip)_4_] a transference number of *t_+_* = 0.31 (cf. *t_+_* = 0.2–0.4 for common LIB electrolytes^[^ ^49^ ^]^) was determined reflecting the metal cations to be less mobile by the stronger solvent interaction than the [B(hfip)_4_]^−^ anions. According to previous studies, the transference number of the 0.2 m Mg[B(hfip)_4_]_2_/G1 electrolyte is even lower (*t_+_* = 0.185).^[^ ^50^ ^]^ This further backs above assumption that the charge is largely carried by the anion.

### 2.4 Polarization

**Figure 5:**
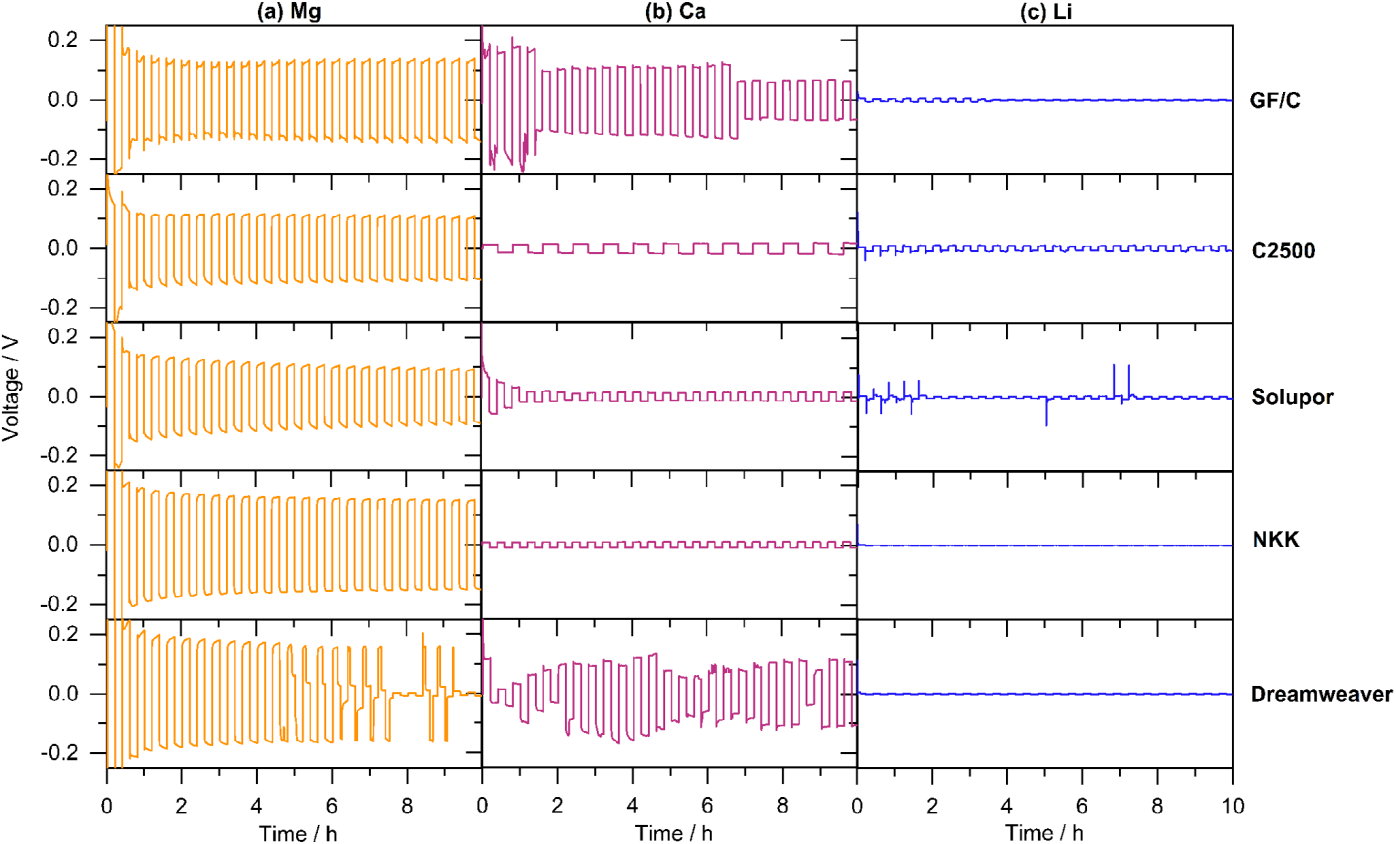
Polarization of a) Mg|Mg, b) Ca|Ca, and c) Li|Li cells comprising a 0.2 m M[B(hfip)_4_]_n_ / G1 electrolyte at 1.0 mA cm^−2^ applying different separators. In case of GF/C 150 µl, in other cases 50 µl electrolyte volume was used.

Further, it was investigated how the transport properties affect the electrochemical performance in Mg, Ca, and Li symmetrical cells with different separators. Their voltage trend during polarization is depicted in **Figure** **5**. Overpotentials therein either arise due to kinetic limitations caused by i) activation (e.g. electron transfer, chemical reactions, polarization, desolvation, adsorption), ii) concentration (e.g. depletion of charge carriers at the electrode surface through diffusion limitations), or iii) resistance (e.g. counter EMF at junctions like electrode surfaces and interfaces).

Despite the highest ionic conductivity of the electrolyte (Figure 1), the Mg|Mg cells exhibit the highest polarization potentials—independent of the separator material (Figure 5a). This points to the bulk electrolyte properties (*σ*, *E_A_*, D_±_ and *t*_+_) being less important than the interfacial processes (*R_ct_*, *R_SEI_*, and J*) for this metal anode. This is backed by a larger applied electrolyte volume (in multiple separator layers) only hardly increasing the polarization potential but drastically enhancing the cycling stability (Figure S8, Supporting Information). This is in line with recent findings of the cycle life being proportional to the separator thickness due to non‐uniformly distributed magnesium deposits intruding the separator.^[^ ^51^ ^]^ It is assumed that a large electrolyte reservoir is beneficial for the (ongoing) SEI formation to avoid ion depletion. A stable, ion‐conductive SEI is reported in the presence of Li salts by applying Mg/Li hybrid electrolytes.^[^ ^20^, ^52^ ^]^ GF/C shows superior cycling stability (Figure S9, Supporting Information)—especially compared to popular C2500—hence it is most commonly applied in Mg research. However, the interpretation of the sudden cell failure remains uncertain as the post mortem impedance spectra of a Mg|C2500|Mg cell (Figure S10, Supporting Information) does not indicate a short circuit, but impedances on the level with Ca and Li spectra—hence a soft short circuit due to mossy‐grown Mg metal is supposed.

In contrast, symmetrical Ca cells feature significantly lower overpotentials when replacing thick GF/C with thin polyolefin or cellulose separators (Figure 5b), reflecting a dominant bulk electrolyte influence over the interfacial processes, which is in line with lower *R_ion_* values for Celgard, Solupor, and NKK (Figure S6, Supporting Information). This is originated in either i) a lower energy barrier for dissociation/desolvation of Ca^2+^ than Mg^2+^, which was indeed reported to be significantly smaller than Mg^2+^ considering TFSI^−^ anion and EC solvent^[^ ^33^ ^]^ or ii) a SEI on Ca metal, which offers sufficient ionic bulk conductivity or high porosity with fast liquid pathways—in contrast to Mg metal. As the EIS investigation (see subsequent chapter) reveals similar SEI resistances, latter is unlikely.

**Table 3:** Recommendation of suitable separator materials for (practical) Mg, Ca and Li metal battery systems. Note, in case of Mg, thick glass fiber sheets still represent the benchmark separators in terms of cycling stability (e.g. Whatman GF/C).

	Mg	Ca	Li
Tortuosity	Low	Low	Low
Porosity	High (not impeding Mg^2+^ diffusion)	Low (minimize electrolyte volume)	Low (prevent short circuits, minimize electrolyte volume)
Pore size	Large (not impeding Mg^2+^ diffusion)	Flexible (no short circuit risk)	Small (prevent short circuits)
Chemical compo‐sition	Polyolefin (less Mg intrusion)	Flexible (cellulose provides better wetting)	Flexible (cellulose provides better wetting)
Example	Solupor 3P07A	NKK TBL4620	Celgard 2500

Generally, Li|Li cells show very low polarization potentials (< 5 mV, Figure 5c) and impedances (< 60 Ω, Figure S11a, Supporting Information). However, with separators exhibiting a loose fiber network with large porosity and pore size lithium faces early short circuits due to facilitated dendrite growth. While GF/C withstands some stripping/plating cycles due to large thickness (>200 µm), thin separators with large porosity fail instantly (Solupor, NKK, Dreamweaver). The short circuit is verified to be induced electrochemically rather than due to inaccurate cell assembly (Figure S11b, Supporting Information). The apparently too low crossover current is either originated in the low salt concentration (0.2 m) or the sluggish surface diffusion—a known shortage of Li metal anodes. As Mg and Ca do not suffer from early cell failure with the same low cation concentration (yet doubled anion concentration) in the electrolyte, latter is more likely. This confirms the benefit of earth alkaline metals in terms of lower tendency for dendrite formation and consequent improved safety. In contrast to the other thin separators, C2500 is capable to withstand the Li dendrite breakthrough (Figure S11c, Supporting Information) due to the strong polymeric network only containing micropores (64 nm). Hence, Celgard 2500 is among the most commonly used separators in Li metal battery research.

**Figure 6:**
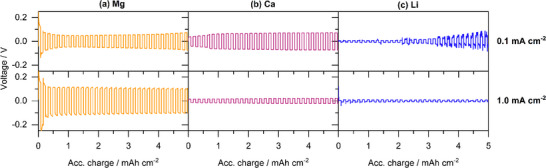
Polarization of symmetrical a) Mg, b) Ca, and c) Li cells comprising a 0.2 m M[B(hfip)_4_]_n_ / G1 electrolyte at 0.1 and 1 mA cm^−2^ applying a C2500 separator.

The polarization potential is obviously closely related to the applied current density—whereby the interfacial resistances (charge transfer and SEI) are expected to decrease and the ohmic resistance (mainly electrolyte) to increase with increasing current. Due to the dominant ohmic cell resistance, the overall polarization potential is usually increasing with increasing current which holds for Mg independent of the applied separator (**Figure** **6**a: C2500; Figure S12a, Supporting Information: GF/C). Interestingly, Ca exhibits the opposite trend—namely decreasing polarization potential at higher currents (Figure 6b; Figure S12b, Supporting Information). This further indicates the slow desolvation or interfacial processes in Mg case, while there is no kinetic limitation for Ca. In fact, a higher current probably induces a larger number of nuclei and consequently larger electrochemically active Ca surface. In case of Li, the polarization overpotential hardly indicates a current dependency, i.e. changes interfacial and ohmic resistances offset each other.

To understand the interplay of interfacial effects and the electrolyte properties that govern charge transport and transfer in these three metal systems, the next chapter discusses the symmetrical metal anode impedance response both at open circuit voltage (OCV) and under current for a range of separators and current densities.

### 2.5 Electrochemical Impedance Spectroscopy

#### 2.5.1 Adsorption Layer Formation during OCV

**Figure 7:**
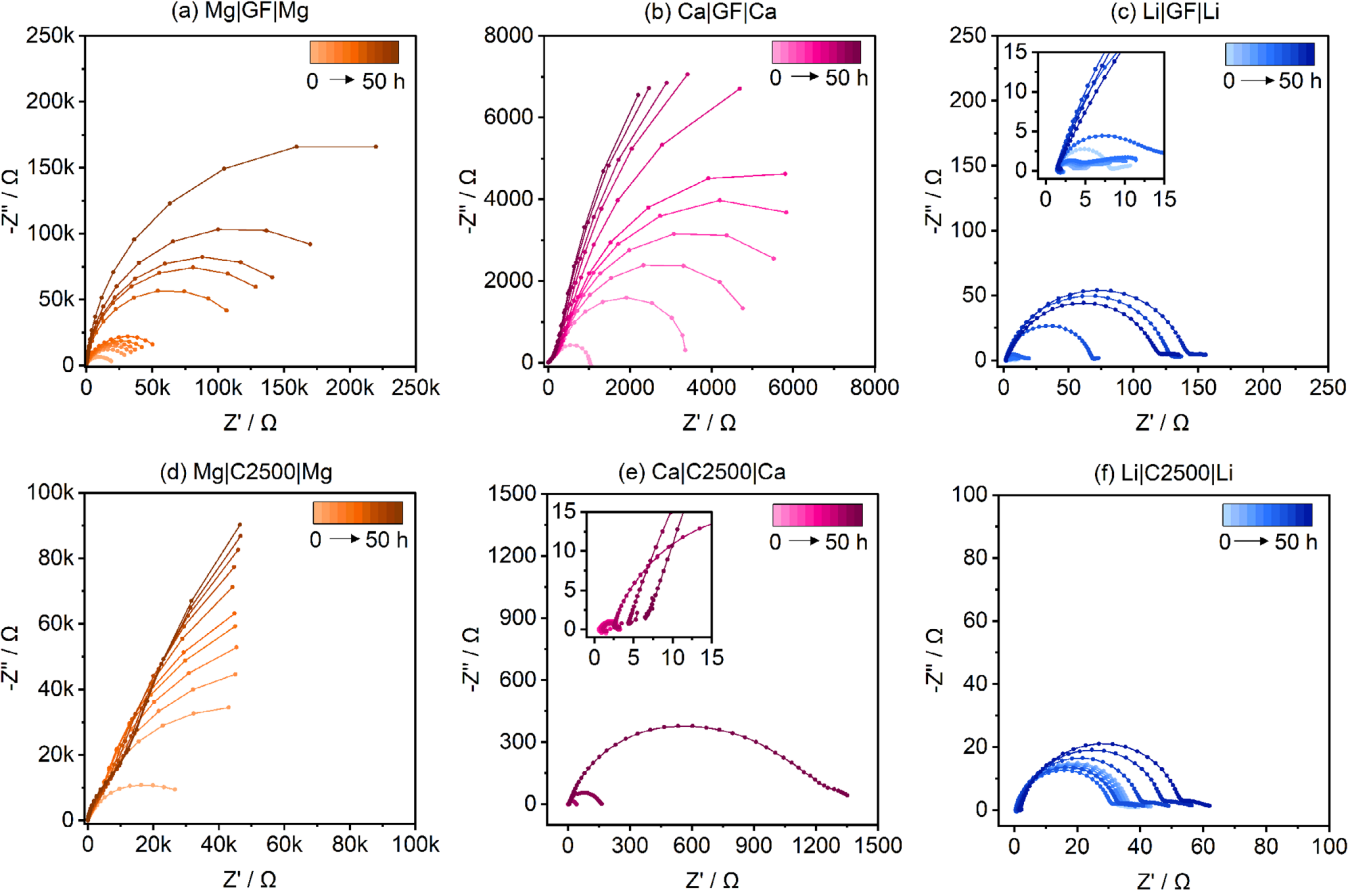
Impedance spectra evolution during 50 h OCV in Mg|Mg, Ca|Ca and Li|Li symmetrical cells with two layers of GF/C ((a–c) 250 µl) and one layer of C2500 ((d,e) 50 µl), respectively. The corresponding Bode plots are depicted in Figure S14 (Supporting Information).

To investigate the non‐faradaic interfacial processes over time, potentiostatic EIS measurements are performed during 50 h OCV after cell assembly. The Nyquist plots of the Mg|Mg, Ca|Ca, and Li|Li cells are shown in **Figure** **7**a–c, respectively. Known from previous studies, magnesium metal is prone to form a passivating layer consisting of loosely packed, adsorbed solvent and salt molecules during non‐current conditions, which is therefore commonly called “adsorption layer”.^[^ ^53^, ^54^, ^55^ ^]^

The adsorbed species provoke a large interfacial resistance of several hundred kΩ (Figure 7a,d). Independent of applying C2500(Figure 7d) or GF/C (Figure 7a), magnesium metal exhibits a drastic increase in impedance—yet with lower characteristic frequency in case of C2500 (Figure S14d, Supporting Information). In contrast, the adsorption layer formation on Ca metal depends on the electrolyte volume and/or the separator structure and chemical composition. With GF/C separator, Ca metal shows the same impedance increase as Mg metal, exhibiting one order of magnitude lower values (Figure 7b)—yet also lower characteristic frequencies (Figure S14b, Supporting Information). As pointed out above, with C2500 (Figure 7e), Ca shows similar behavior than Li (Figure 7c,f)—namely only one distinguishable process with increasing impedance. Yet, in the Ca system the process exhibits a significant shift to lower characteristic frequencies (Figure S14e, Supporting Information), while lithium hardly features any changes in characteristic frequency (Figure S14f, Supporting Information)—which points to an electrochemically active lithium surface. However, the increase in impedance of Li|Li cells over time also indicates a non‐faradaic surface layer formation, which is more pronounced with larger electrolyte volume (Figure 7c,f). Note, that an EIS amplitude of 5 mV will disturb the Li system in greater manner than the Mg and Ca system due to the significantly lower polarization potential (cf. Figure 5), which might be sufficient to induce a SEI rather than an adsorption layer formation. It was shown that even the lowest excitations would lead to stripping/plating in the Li system.^[^ ^56^ ^]^ Due to non‐distinguishable processes, the charge transfer and ion diffusion through the SEI layer have to exhibit similar time constants.

**Figure 8:**
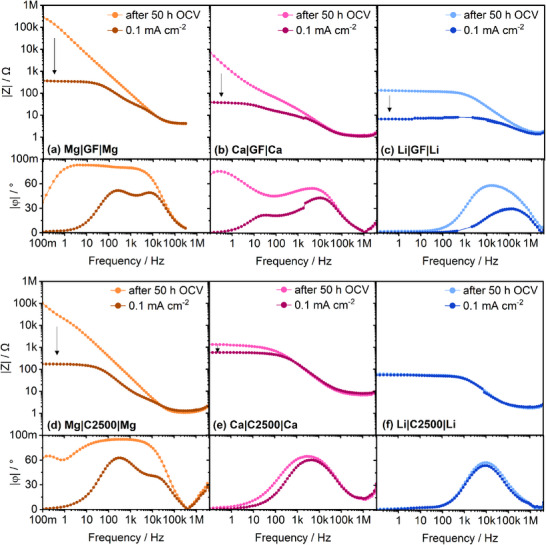
Comparison of the final impedance spectra after 50 h OCV and the first impedance spectra during polarization with 0.1 mA cm^−2^ in Mg|Mg, Ca|Ca and Li|Li symmetrical cells with two layers of GF/C ((a–c) 250 µl) and one layer of C2500 ((d—f) 50 µl), respectively. The corresponding Nyquist plots are depicted in Figure S15 (Supporting Information).

Interestingly, for Mg and Ca, by applying a polarization current of 0.1 mA cm^−2^, the adsorbed electrolyte species instantly desorb resulting in low overpotentials. Therefore, to avoid the build‐up of such adsorption layer during EIS measurements, galvanostatic EIS with *I_eis_* = *I_pol_* (5 mV amplitude) were performed. Due to this “operando EIS”, the high‐ohmic adsorption layer in Mg and Ca cells is indeed mitigated, and the practical impedance during cell operation is recorded (**Figure** **8**a,b,d). Note, that the adsorption layer on Mg anodes forms reversibly during polarization‐intermittent OCV phases (Figure S16, Supporting Information), yet with lower values, while it becomes absent in Ca anodes after polarization at higher current densities (Figure S17, Supporting Information), which is explainable by a dense SEI formation hindering the adsorption of electrolyte species.

Even the Li|Li cell comprising GF/C separator shows a decrease in impedance, which either points to i) a removed adsorption layer or ii) a beneficial SEI formation with superior ion conductivity. Due to the characteristic frequency hardly being altered, latter is more likely (Figure 8c). While the Mg|Mg cells comprising C2500 and GF/C separator show similar behavior (Figure 8d; Figure S18, Supporting Information), the Ca|Ca and Li|Li cells significantly differ as the spectra hardly change upon polarization with C2500 separator (Figure 8e,f). Thus, either no adsorption layer is formed, the SEI formation took place chemically or its formation is induced by the EIS measurements during OCV. In any case, the impedance also slightly increases in subsequent non‐faradaic conditions (Figures S19 and S20, Supporting Information).

**Figure 9:**
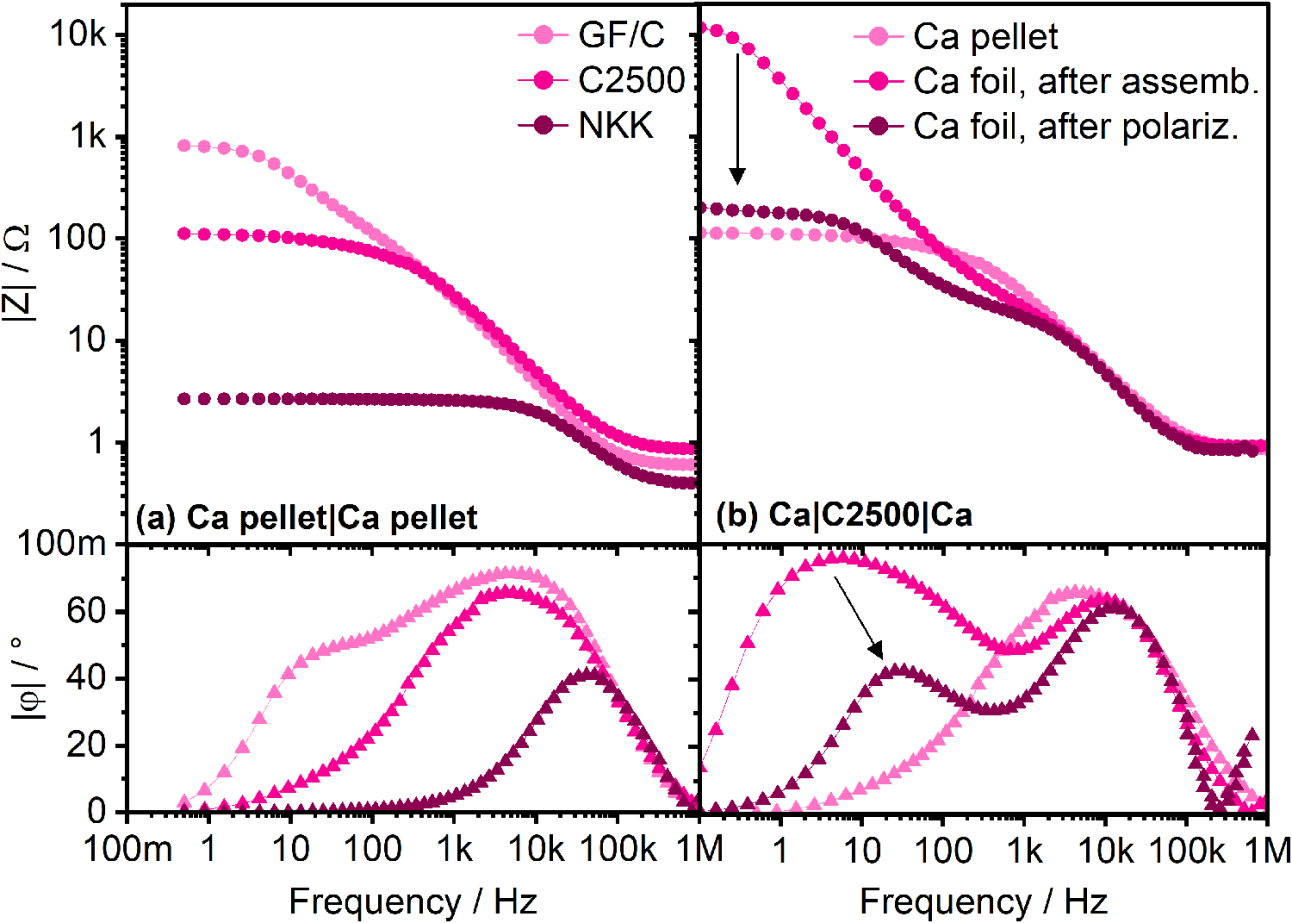
Impedance spectra of Ca|Ca cells comprising a) pellet electrodes and different separators and b) different Ca electrodes and C2500 separator (pot.stat., 5 mV).

As depicted above, in case of Ca|Ca cells, there is a huge difference in impedance comparing GF/C with thin separators—even directly after cell assembly (**Figure** **9**a). The contribution in the low‐frequency region (LF) of the Ca|GF|Ca cell can be partly explained by the formation of an adsorption layer. However, this second process is also present when applying Ca foil instead of the Ca pellet—yet with declining impedance and smaller time constant after polarization (Figure 9b). It is therefore assigned to a surface process, most probably charge transfer, which is delayed and not anymore superimposed by the SEI processes by i) a larger surface area (roughened Ca surface after polarization) and ii) larger electrolyte volume (longer diffusion length and mass‐transport limitation).

#### 2.5.2 Resistance Evolution during Polarization

**Figure 10:**
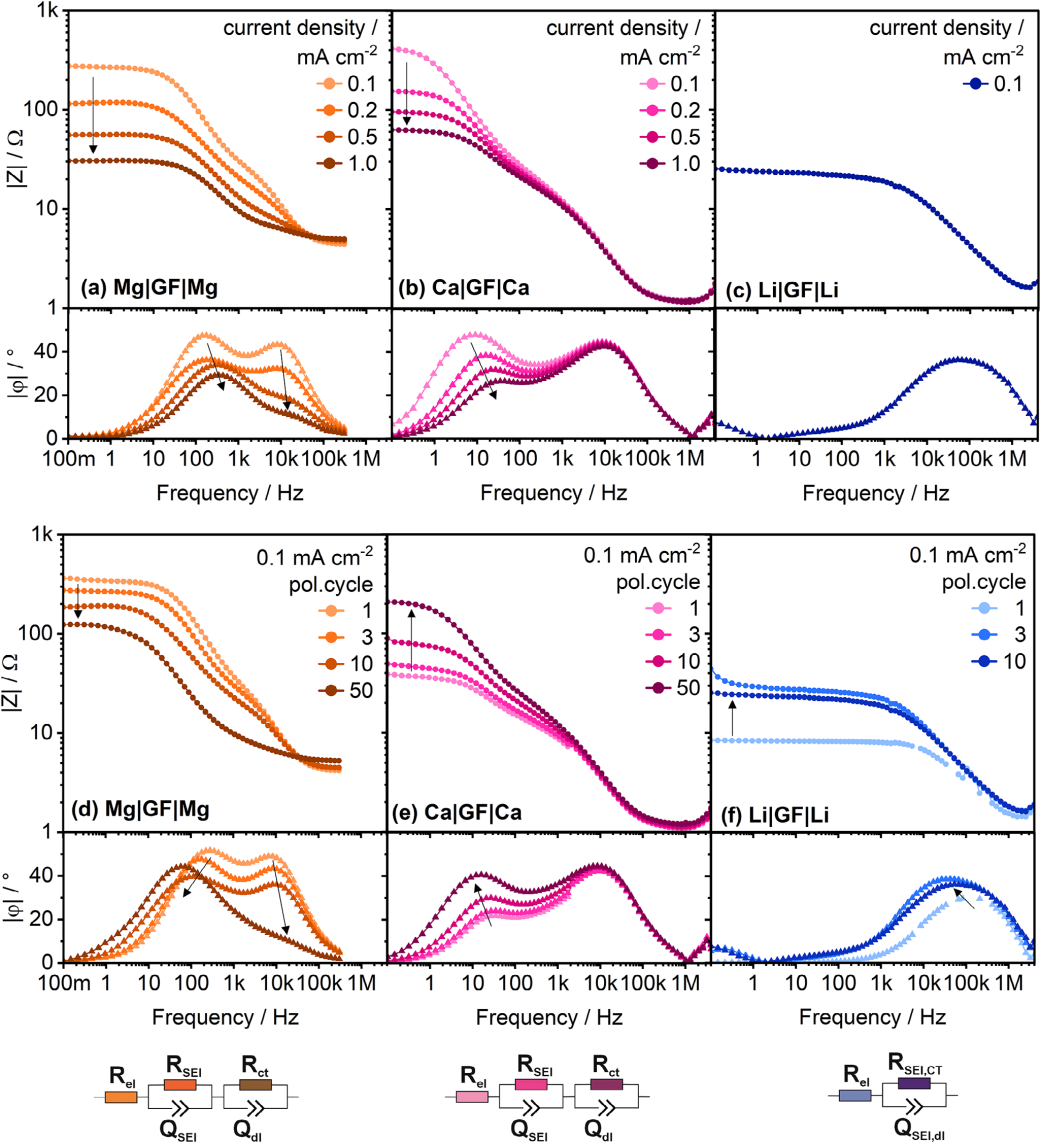
Impedance spectra and corresponding ECM of Mg|Mg, Ca|Ca and Li|Li cells during a–c) polarization at different current densities and d–f) polarization at 0.1 mA cm^−2^ (2x GF/C, 250 µl).

To gain further insight into the surface processes, the impedance spectra collected during polarization at different current densities and with proceeding cycle number are evaluated (**Figure** **10**d–f). As pointed out above, glass fiber is not capable to withstand the lithium dendrite penetration (despite applying two GF/C layers), hence only a current density of 0.1 mA cm^−2^ could be measured (Figure 10c). The impedance spectra of analogous cells with C2500 separator are depicted in Figure S21 (Supporting Information) for comparison.

In both, Mg|Mg and Ca|Ca cells two distinct processes are present with the overall impedance decreasing with increasing current density *i* (Figure 10a,b). Further, the MF process decreases in impedance and phase angle with simultaneous peak shift to higher frequencies. Hence, it is assigned to the charge transfer resistance *R_ct_*, and double‐layer capacitance *C_dl_* due to its definition:

(5) $$R_{ct}=\frac{RT}{nFi},\;\;C_{dl}=\frac{\varepsilon A}{d_{dl}}=const.$$

(6) $$f_{c}=\frac{1}{R_{ct}C_{dl}},f_{c}\propto i$$

**Figure 11:**
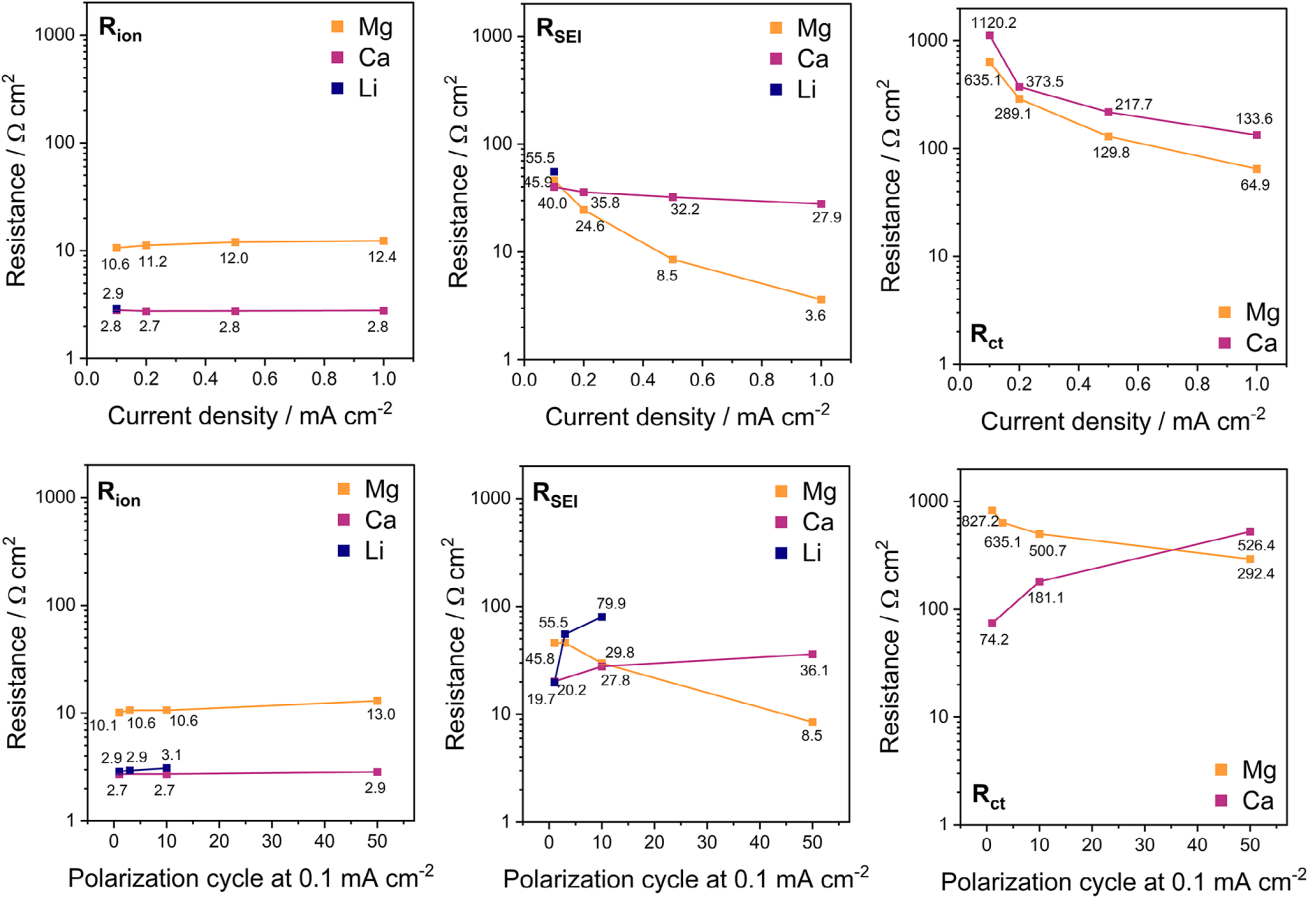
Fitted resistance values corresponding to the spectra in Figure 10. The fit errors were included yet too small be visible herein.

Due to the thickness of the electric double‐layer *d_dl_* being independent from the current density as it is determined by the size of the solvent molecules, *C_dl_* remains constant and consequently the characteristic frequency *f_c_* increases linearly with *i*, which is consistent with the shift in phase angle (Figure 10a,b). Further, the HF process is assigned to the Mg^2+^/Ca^2+^ conduction through the solid electrolyte interphase *R_SEI_*. Straightforward, an ECM comprising two R‐CPE elements in series to an ohmic resistance is applied to extract distinct resistance values. In contrast, only a single R‐CPE element is used—yet only HF‐region (< 5 kHz) is fitted. Note that, in either case the projected electrode area (2.545 cm^2^) is considered to determine the areal resistance values—also for the rough/microporous Ca pellet‐yet the actual electrochemically active surface area is unknown (**Figure** **11**).

While most of the resistance values feature a similar trend for the different metal systems, the evolution of *R_SEI_* and *R_ct_* in Mg|Mg cells significantly differ by decreasing with ongoing polarization. This is originated in either i) an enhanced ion diffusion through the SEI—which is unlikely due to the SEI components being known to exhibit low Mg^2+^ conductivity—or ii) the surface area being increased due to non‐planar Mg deposition resulting in rough Mg deposits. Due to those additional reactive Mg metal sites, continuous electrolyte decomposition is likely, which causes the electrolyte resistance *R_ion_* to slightly increase, whereas it remains constant in the Ca|Ca cell throughout the polarization with no signs of degradation or cell leakage. The linear relation of *R_ct_* to *i*—especially in case of Mg—confirms above assignment (Equation (5)). In case of Ca, the increase of *R_ct_* at higher polarization cycles is assigned to the charge transfer being mass‐transport limited as the diffusion through the SEI is increasingly impeded (simultaneously increasing *R_SEI_*). Nonetheless, the in situ formed SEI is still superior to the native surface layers (mostly CaO), with the non‐scraped Ca metal showing instable open cell potential and poor electrochemical activity (Figure S22 and S23, Supporting Information). Interestingly, the adsorption layer forms in either case.

Note, that the lower anion concentration of the 0.2 m Li[B(hfip)_4_] electrolyte compared to the divalent counterparts might not only affect the electrolyte transport properties as discussed above, but also its interfacial stability. At high concentrations, increased ion‐pairing can promote anion decomposition near the cation during plating, leading to a substantially inorganic SEI. In contrast, lower concentrations favor solvent decomposition, resulting in a mainly organic SEI. These compositional differences influence SEI stability, ion conductivity, dendrite formation, and cycling performance. However, the 0.4 m Li [B(hfip)₄]/G1 electrolyte remains below the solubility limit (approx. 0.6 M),^[^ ^2^ ^]^ and the Raman spectra show no significant increase in ion‐pairing over the 0.2 m electrolyte. Furthermore, already with 0.2 m electrolyte concentration, Li shows lower SEI resistance and impedance than its divalent counterparts.

## 3 Conclusion

The present study provides a comprehensive comparison of magnesium, calcium and lithium B(hfip)_4_‐based electrolytes and metal anodes in terms of electrolyte transport properties including the conductivity, viscosity, diffusion coefficient and transference number, and anode‐related interfacial processes such as the charge transfer and SEI diffusion. In this context, five different separators are selected to investigate their suitability for the specific battery system.

To this end, the apparent tortuosity was surprisingly found to be cation‐dependent, which is assumed to originate from the different charge density and size of the cations (Mg^2+^, Ca^2+^, Li^+^), resulting in altered solvation shells that affects their diffusion in the porous separator media. This, however, is counter‐intuitive as the porosity and pore size of the tested separators are indeed beyond the even highly solvated ions in these systems. While the observed differences in tortuosity between cations are negligible in the case of Whatman GF/C, which features large pores and high porosity, they become substantially more pronounced in tortuous separators—such as Dreamweaver DW25—pointing to the stronger influence of the pore structure on the ion‐specific transport behavior. Interestingly, the ambipolar diffusion coefficient of the Ca[B(hfip)_4_]_2_ electrolyte is found to be higher than that of its Mg and Li counterparts, which is attributed to the more effectively shielded charge of Ca^2^⁺ by its large solvation shell, thereby reducing ion‐pair formation. Thus, the mobility is enhanced with the [B(hfip)_4_]^−^ anion being the main charge carrier as the cation transference number is rather low—at least in case of Li (t_+_ = 0.31).

To compare the stripping–plating behavior in symmetrical cells, polarization experiments reveal distinct trends across the three metal systems. Mg exhibits the highest overpotential—independent of the applied separator, suggesting that interfacial processes are the rate‐limiting factor. Herein, cycling stability is strongly affected by separator thickness and electrolyte volume, with thinner separators and lower electrolyte volumes proving detrimental. Accordingly, separators with large porosity and pore sizes are beneficial, as they help mitigating ion depletion at the interface and potentially promote more homogeneous Mg deposition. Among the tested separators, polyolefin types—despite differences in porosity—consistently deliver lower overpotentials and longer cycle life than their cellulose‐based counterparts in the Mg system, suggesting that the chemical nature of the separator may also influence the deposit morphology.

In contrast, Ca cells exhibit a different behavior: thin separators, regardless of being polyolefin‐ or cellulose‐based, result in stable cycling at very low overpotentials (<20 mV at 1 mA cm^−2^). As the ohmic resistance correlates (linearly) with the separator thickness and tortuosity, very thin and low‐tortuous separators are recommended. Smaller pores (<100 nm) are found to enhance the cycling stability, while porosity, and chemical composition are otherwise less critical. Interestingly, a higher current density results in lower polarization overpotentials (independent of the separator), which is assigned to the formation of more calcium nucleation sites and consequently larger deposition surface area.

Lithium generally exhibits low polarization overpotentials, but early cell failure with highly porous separators due to short‐circuiting, which points to the different depositing and growth mechanisms of mono‐ and divalent systems. **Table** **3** summarizes above findings to provide a recommendation regarding suitable separator materials for each specific battery systems.

Further details about the interfacial processes at the metal anodes are gained by EIS measurements revealing Ca to exhibit an interfacial behavior that at times resembles Li and at other times mirrors Mg response. For instance, while a high‐ohmic adsorption layer—analogous to that observed in Mg cells—forms in Ca cells with large electrolyte volume (e.g. GF/C), it is absent under low electrolyte volume conditions (e.g. C2500). In contrast, Li does not exhibit such high‐ohmic layer—neither with large or low electrolyte volume—showing a rather fast CT whose time constant overlaps with the SEI process. By applying “operando EIS” during polarization, essential for bivalent systems, the adsorption layer is mitigated and the SEI and charge transfer resistance become eventually visible in the impedance response of Mg and Ca cells. However, in the case of thin separators, Ca exhibits similar behavior to Li, i.e. overlapping of SEI and charge transfer process, as latter is not being decelerate by mass‐transport. Concomitant with the polarization experiment the relaxation time of the CT process in the Mg system remains independent of separator thickness and consistently appears at higher frequencies compared to the SEI.

Overall, this study reveals fundamental differences in electrolyte transport and interfacial processes of Mg, Ca, and Li metal batteries—and the influence of the separator therein. Especially in case of calcium, the separator choice significantly alters the charge transfer characteristics and electrochemical behavior. With thin separators showing promising performance, they are strongly recommended for future studies to identify limitations already in the early research state of the Ca metal battery. As it shares similarities to both its Li and Mg counterparts, the community might benefit from their research to accelerate its own development, which is worthwhile due to its promising performance along with its enhanced sustainability and safety over lithium.

## Conflict of Interest

The authors declare no conflict of interest.

## Supporting information



Supporting Information

## Data Availability

The data that support the findings of this study are available from the corresponding author upon reasonable request.
