# Supplementary material for: Electrolyte Transport Parameters and Interfacial Effects in Calcium Metal Batteries: Analogies and Differences to Magnesium and Lithium Counterparts
Source: Adv Sci (Weinh). 2025 Jun 25;12(33):e06498. doi: 10.1002/advs.202506498 (PMC12412554; doi:10.1002/advs.202506498)
Supplement: Supplementary file 1 — Supporting Information [file ADVS-12-e06498-s001.pdf]

ADVANCED SCIENCE
Open Access

Supporting Information

for *Adv. Sci.*, DOI 10.1002/advs.202506498

Electrolyte Transport Parameters and Interfacial Effects in Calcium Metal Batteries: Analogies and Differences to Magnesium and Lithium Counterparts

Joachim Häcker, Tobias Rommel, Laurin Rademacher, Sibylle Riedel, Zhirong Zhao-Karger, J. Alberto Blázquez, K. Andreas Friedrich and Maryam Nojabaee**

Supporting Information

Electrolyte Transport Parameters and Interfacial Effects in Calcium Metal Batteries: Analogies and Differences to Magnesium and Lithium Counterparts

Joachim Häcker*,[a], Tobias Rommel[a], Laurin Rademacher[a], Sibylle Riedel[b,c], Zhirong Zhao-Karger[b,c], J. Alberto Blázquez[d], K. Andreas Friedrich[a,e] and Maryam Nojabaee[a]

* Corresponding Author

e-mail: joachim.haecker@dlr.de

[a] Institute of Engineering Thermodynamics, German Aerospace Center (DLR), Pfaffenwaldring 38-40, 70569 Stuttgart, Germany

[b] Helmholtz Institute Ulm (HIU) Electrochemical Energy Storage, Helmholtzstrasse 11, 89081 Ulm, Germany

[c] Institute of Nanotechnology (INT), Karlsruhe Institute of Technology (KIT), Hermann-von-Helmholtz Platz 1, D-76344 Eggenstein-Leopoldshafen, Germany

[d] CIDETEC, Basque Research and Technology Alliance (BRTA), P◦ Miramon, 196, 20014 Donostia-San Sebastian, Spain

[e] Institute of Building Energetics, Thermotechnology and Energy Storage (IGTE), University of Stuttgart, Pfaffenwaldring 6, 70569 Stuttgart, Germany

Anode and electrolyte preparation

The anode preparation was executed in a glovebox under Ar atmosphere (H_2O < 1 ppm, O_2 < 1 ppm). Calcium granules (16 mesh, 99.5 %, Thermo Scientific) were pressed in an 18 mm mold at 4t (154 MPa) to form a Ca pellet of approx. 600 µm thickness (Fig. S1). Magnesium foil (100 µm, 99 %, Gelon) and lithium foil (500 µm, battery grade, MSE Supplies) were cut into 18 mm discs. Ca and Mg are vigorously scraped with a spatula prior to cell assembly to remove the native oxide layer (Fig. S1), while lithium metal was already received in battery grade with shiny, smooth surface.

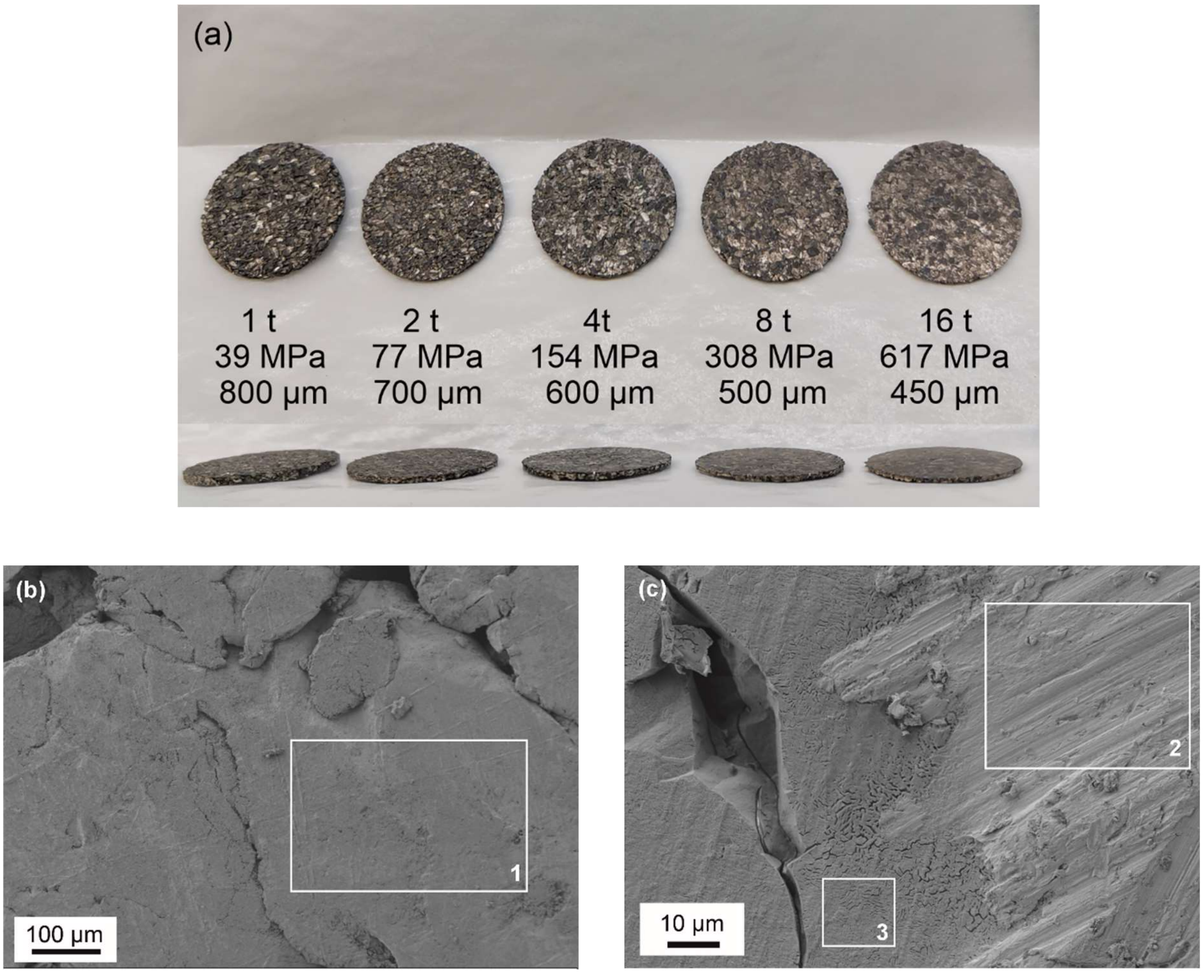

	Spectrum	Ca	O	Cl	F	C	Total
Pristine	1	65.23	31.30	0.20	-	3.26	100.00
Scraped, post mortem	2	91.49	7.53	0.15	0.83	-	100.00
	3	13.53	8.49	-	77.98	-	100.00

Figure S1: Calcium pellets (Ca granules, 16 mesh, 99.5 %, approx. 200 mg, 18 mm diameter): (a) pressed at different pressures (unscraped) with the SEM images and EDX analysis of the (b) pristine and (c) scraped surface post mortem (unwashed from Ca|Ca cell, polarization at 1.0 mA cm^{-2}).

To ensure comparability, an electrolyte system comprising the same anion ($B(hfip)_4^-$) and cation concentration (0.2 M) was selected. The hexafluoroisopropyloxy borate $M[B(hfip)_4]_n$ (Me = Mg, Ca, Li) is synthesized according to previous studies (Fig. S2) [1-3] and dried before use. According to NMR analysis (Fig. S3), the dried salts contain residual G1 molecules, namely $Mg[B(hfip)_4]_2 \cdot 3$ G1, $Ca[B(hfip)_4]_2 \cdot 4$ G1 and $Li[B(hfip)_4] \cdot 3$ G1, resulting in a molecular weight of 1652.53, 1758.42 and 956.23 g mol^{-1}, respectively. After dissolving the salts in G1 (monoglyme, 99.5 %, < 10 ppm H_2O, Acros Organics), the 0.2 M electrolyte solution was purified with a PTFE filter.

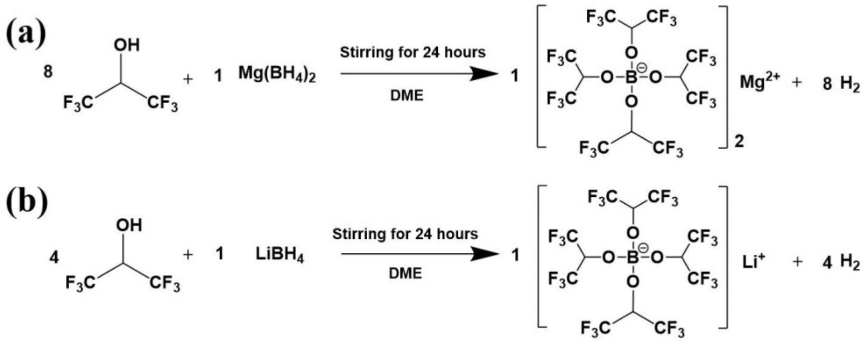

Figure S2: Synthesis routes for the $M[B(hfip)_4]_n$ (M = Mg (n = 2), Ca (n = 2), Li (n = 1)) salts (reproduced from Tang et al.).[3]

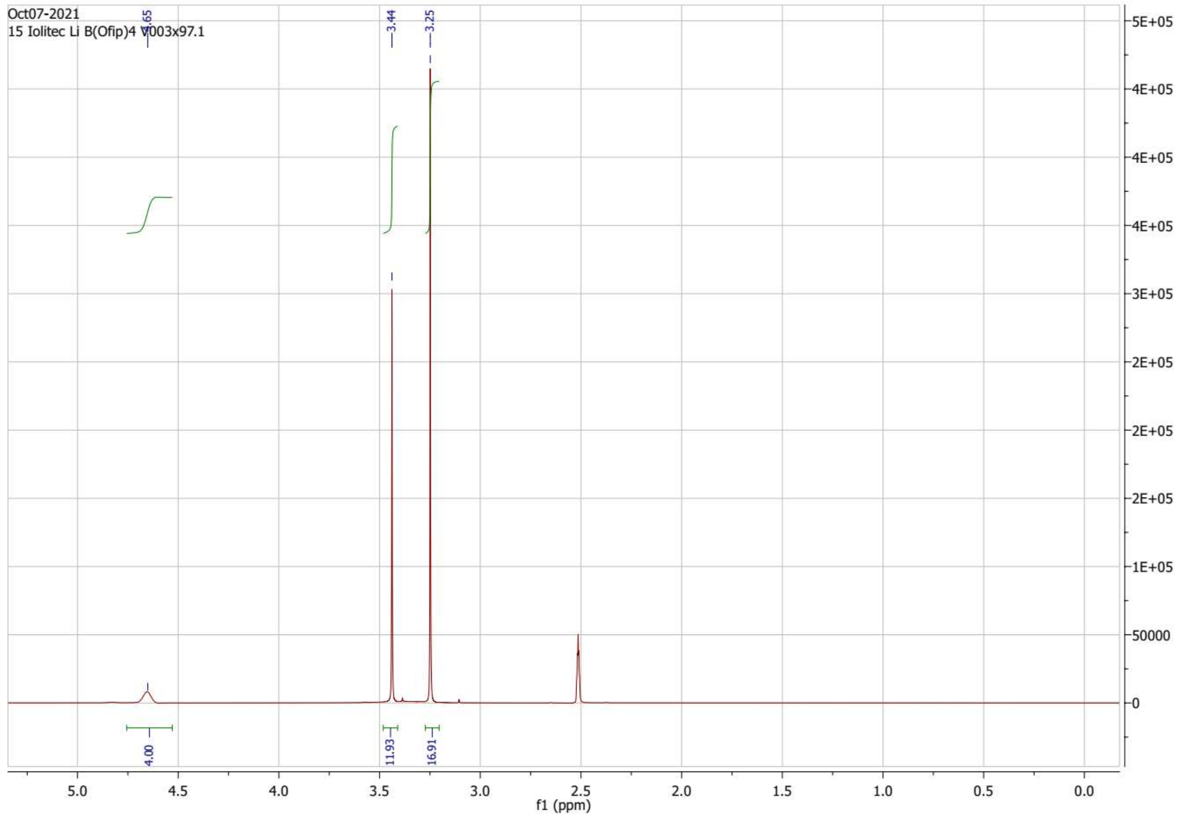

Figure S3: ^{1}H NMR spectrum (DMSO-d6, 500 MHz) concluding a $Li[B(hfip)_4]$ x 3 G1 configuration.

Cell assembly and characterization

Raman spectra of the electrolyte solutions were gained at a Horiba LabRAM with a 532 nm laser and a Synapse detector. Symmetrical Mg|Mg, Ca|Ca and Li|Li cells were assembled in El-Cell PAT-Core setups with different separator materials: Whatman GF/C (260 µm, glass fiber), Celgard 2500 (25 µm, PP), Lydall Solupor 3P07A (28 µm, PE), NKK TBL 4620 (16 µm, cellulose), Dreamweaver Silver 25 (27 µm, cellulose). An electrolyte volume of 150 and 50 µl was added to GF/C and the thin separators, respectively. The diffusion coefficient and transference number were evaluated from galvanostatic pulse polarization ($U_p < 10$ mV) with electrochemical impedance spectroscopy (EIS) prior and after polarization. EIS measurements were performed at a Zahner Zennium device in the frequency range of 1 MHz to 100 mHz with 1-5 mV amplitude. Therein, potentiostatic EIS was utilized during OCV ($U = U_{OCV}$, $I = 0$) and pseudo-galvanostatic spectra were collected during cell operation ($I = I_{pol}$). For the determination of the separator tortuosity, Cu|Cu cells were applied to mitigate charge transfer reactions at the electrode/electrolyte interface.

In either case, the assembled cells are transferred to a climate chamber at 25 °C to undergo galvanostatic polarization at different current densities (0.1, 0.2, 0.5, 1.0 mA cm^{-2}) and intermittent EIS measurements. Further, the separator tortuosity was determined by EIS measurements over time. SEM images of the separators are gained with previous Pt sputtering.

Statistical analysis

The gained data is plotted without further pre-processing (no transformation or normalization). Yet, single point outliers are removed from the impedance spectra to retain their readability. The tortuosity is given in mean values ± SD (n = 2-4). The errors from ECM fitting were included within the resistance plots (Figure 11) yet too small to be visible.

Raman Spectroscopy

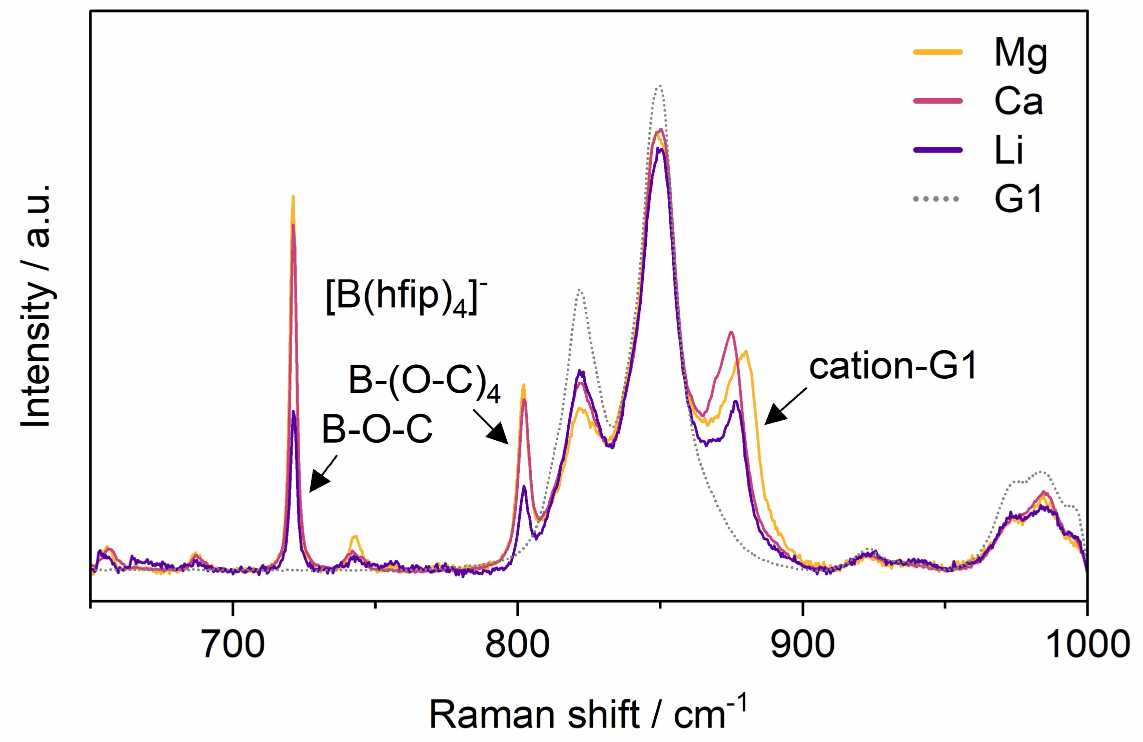

Figure S4: Raman spectra of the 0.2 M $Mg[B(hfip)_4]_2$/G1, 0.2 M $Ca[B(hfip)_4]_2$/G1 and 0.2 M $Li[B(hfip)_4]$/G1 electrolyte, and pure G1.

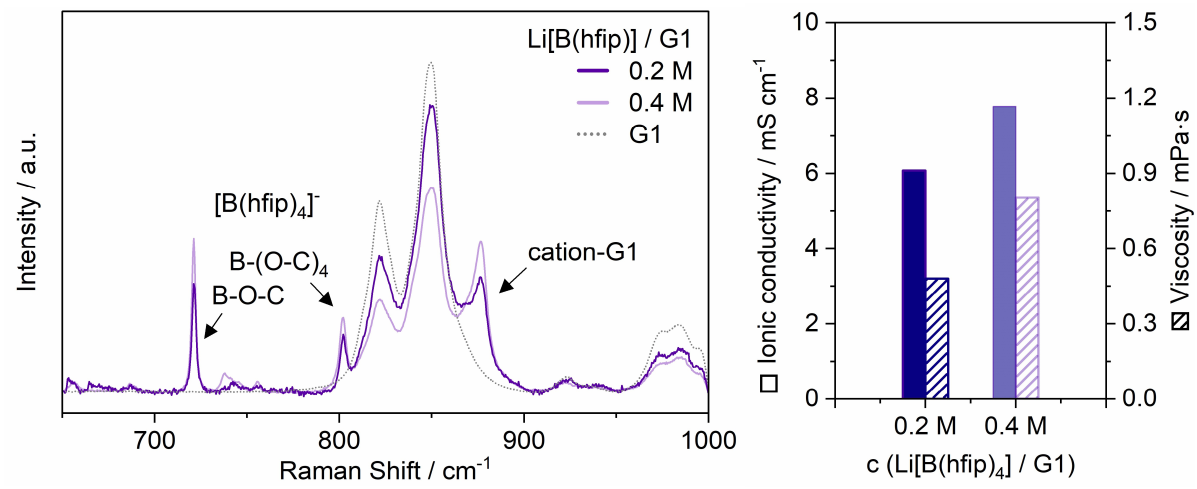

Figure S5: Raman spectra as well as ionic conductivity and viscosity of the $Li[B(hfip)_4]$/G1 electrolyte with different concentrations.

Tortuosity

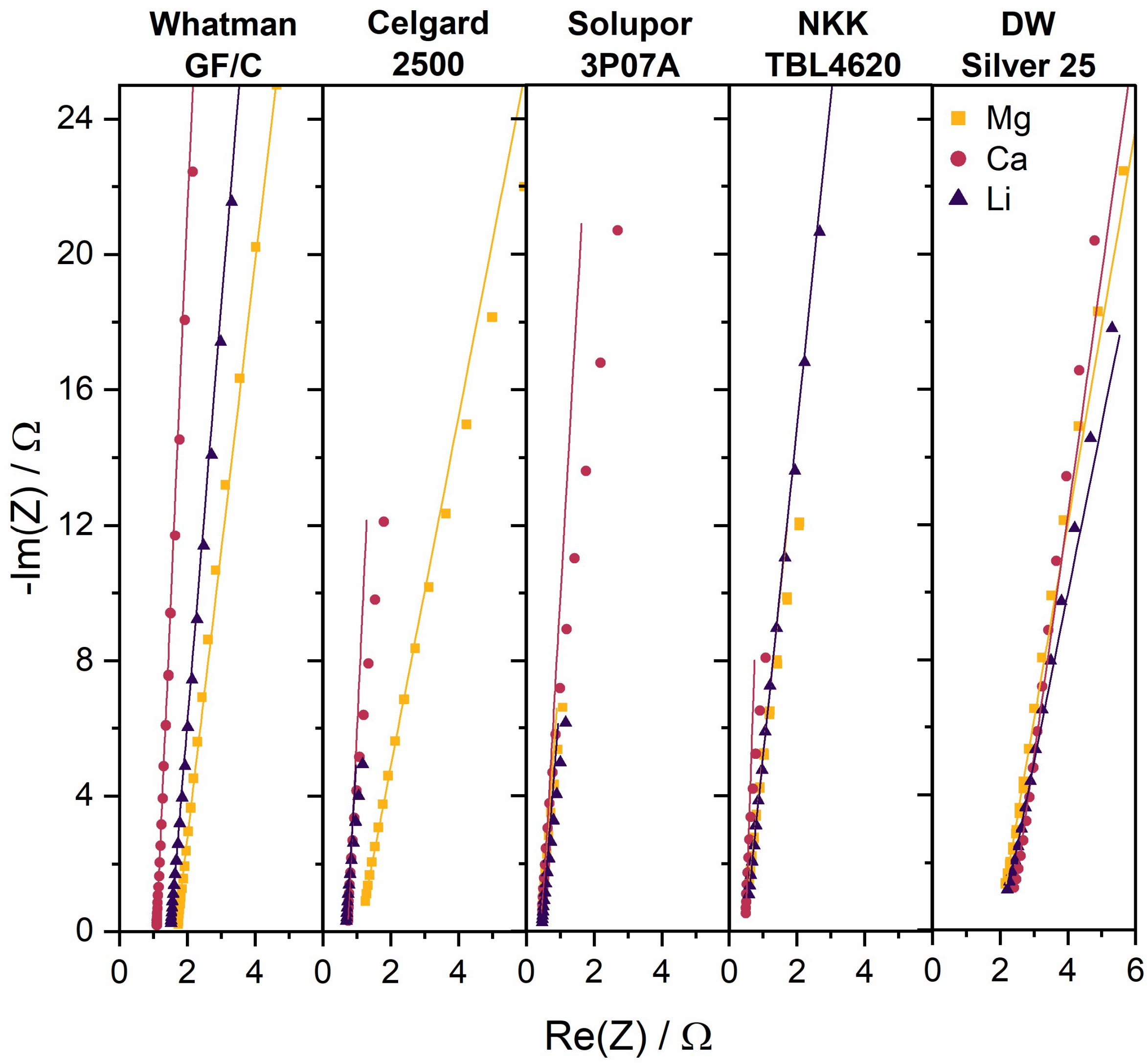

Figure S6: Impedance measurements in Cu/Cu cells and 0.2 M M[B(hfip)$_4$]$_n$ / G1 electrolyte, M = Mg (n = 2), Ca (n = 2), Li (n = 1).

Table S1: Calculated tortuosity and McMullin numbers (std.dev, n=2-4) in different electrolyte systems.

	0.2M $Mg[B(hfip)_4]_2$ /G1		0.2M $Ca[B(hfip)_4]_2$ /G1		0.2M $LiB(hfip)_4$ /G1	
	τ / -	N_M / -	τ / -	N_M / -	τ / –	N_M / -
Whatman GF/C*	1.5 ± 0.2	1.7 ± 0.2	1.2 ± 0.2	1.3 ± 0.2	1.1	1.2
Celgard 2500	4.5 ± 1.0	8.1 ± 1.8	3.7 ± 0.3	6.7 ± 0.6	2.3	4.2
Lydall Solupor 3P07A	4.0 ± 0.02	4.8 ± 0.03	4.2	5.1	2.9	3.5
NKK TBL4620	4.1	5.9	4.2 ± 0.5	6.0 ± 0.7	3.0 ± 0.2	4.3 ± 0.2
Dreamweaver Silver 25	9.6 ± 0.4	17.2 ± 0.7	10.9	19.5	6.4	11.5

* considering the moderate compression in the battery cell a porosity of 90 % and a thickness of 230 µm is assumed (see Figure S7).

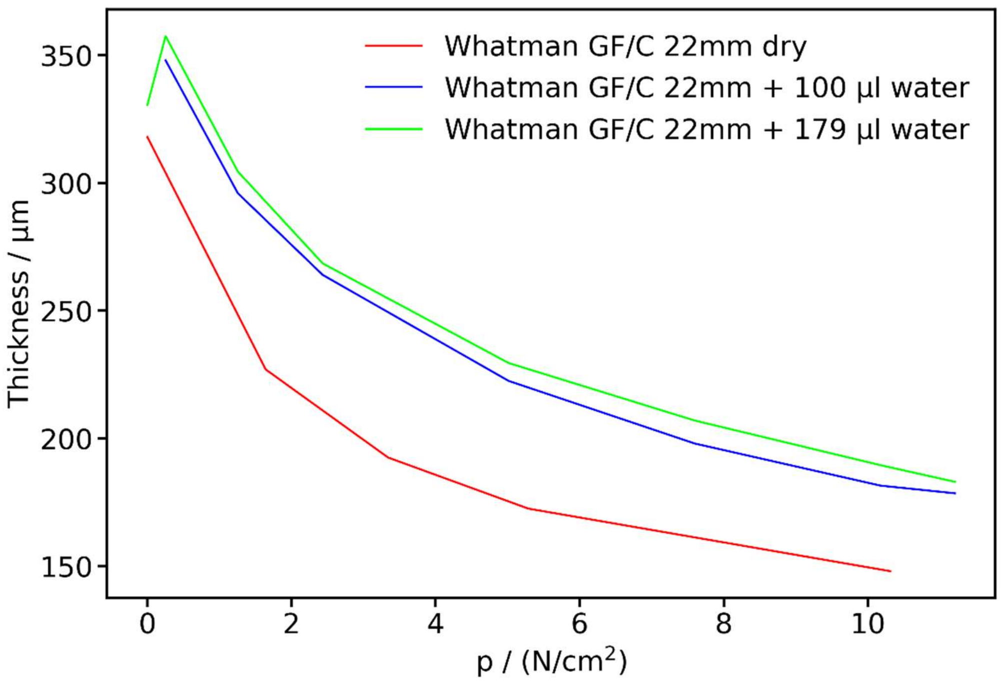

Figure S7: Estimation of the thickness of dry and wet Whatman GF/C under pressure (applied weight). In case of the used EL-PAT-Core a cell pressure of approx. 5 N/cm² and thickness of approx. 230 µm results.

Polarization

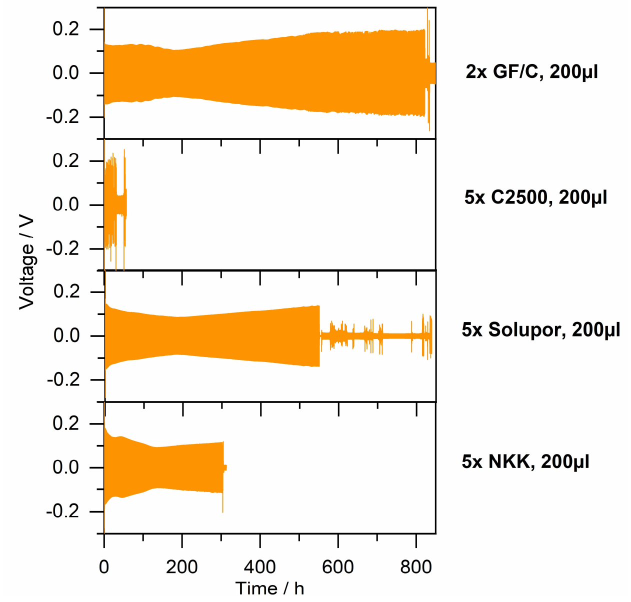

Figure S8: Polarization of Mg|Mg cells at 1 mA cm^{-2} comprising the same volume (200 µl) of 0.2 M Mg[B(hfip)$_4$]$_2$ / G1 electrolyte with multiple separator layers.

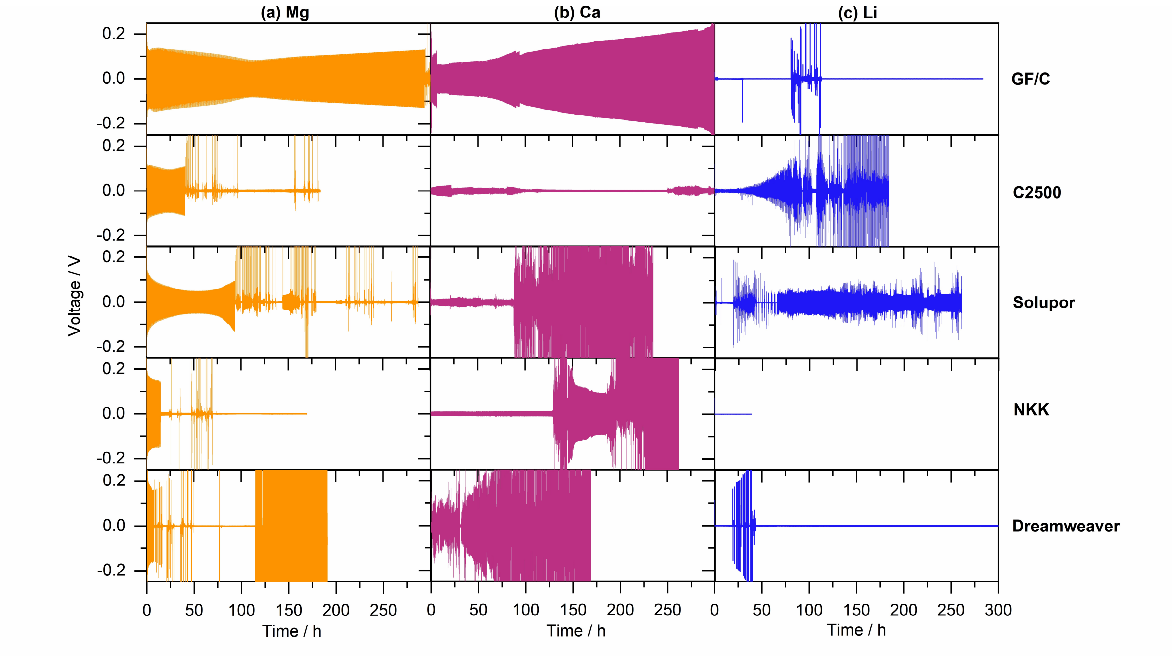

Figure S9: Polarization of (a) Mg|Mg, (b) Ca|Ca and (c) Li|Li cells comprising a 0.2 M M [B(hfip)$_4$]$_n$ / G1 electrolyte at 1 mA cm^{-2} applying different separators. In case of GF/C 150 µl, in all other cases 50 µl electrolyte volume was used.

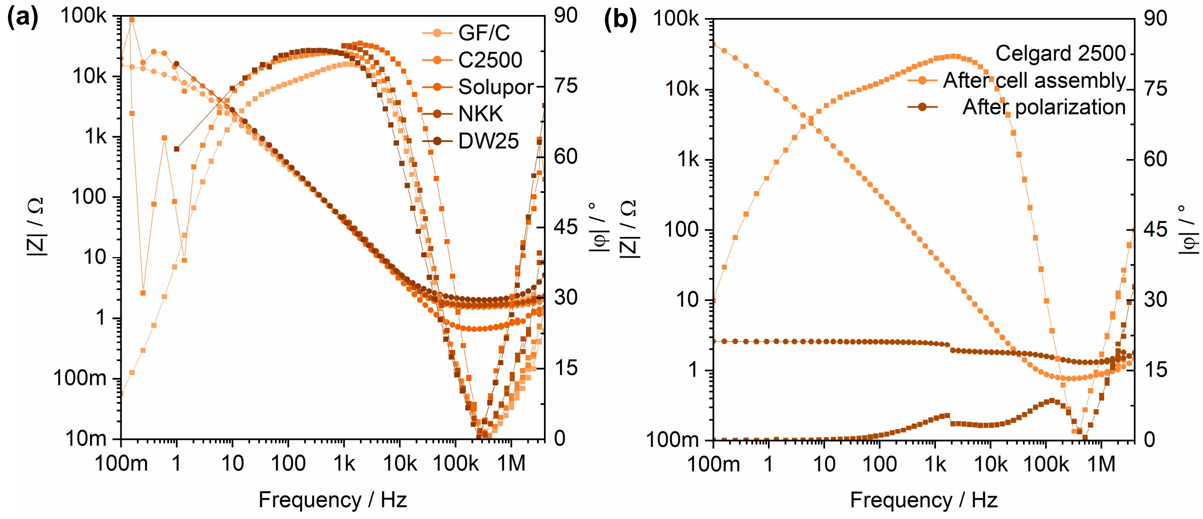

Figure S10: Impedance spectra of a Mg|Mg cell with (a) different separators after cell assembly and (b) Celgard 2500 separator prior and post polarization at 1 mA cm^{-2}.

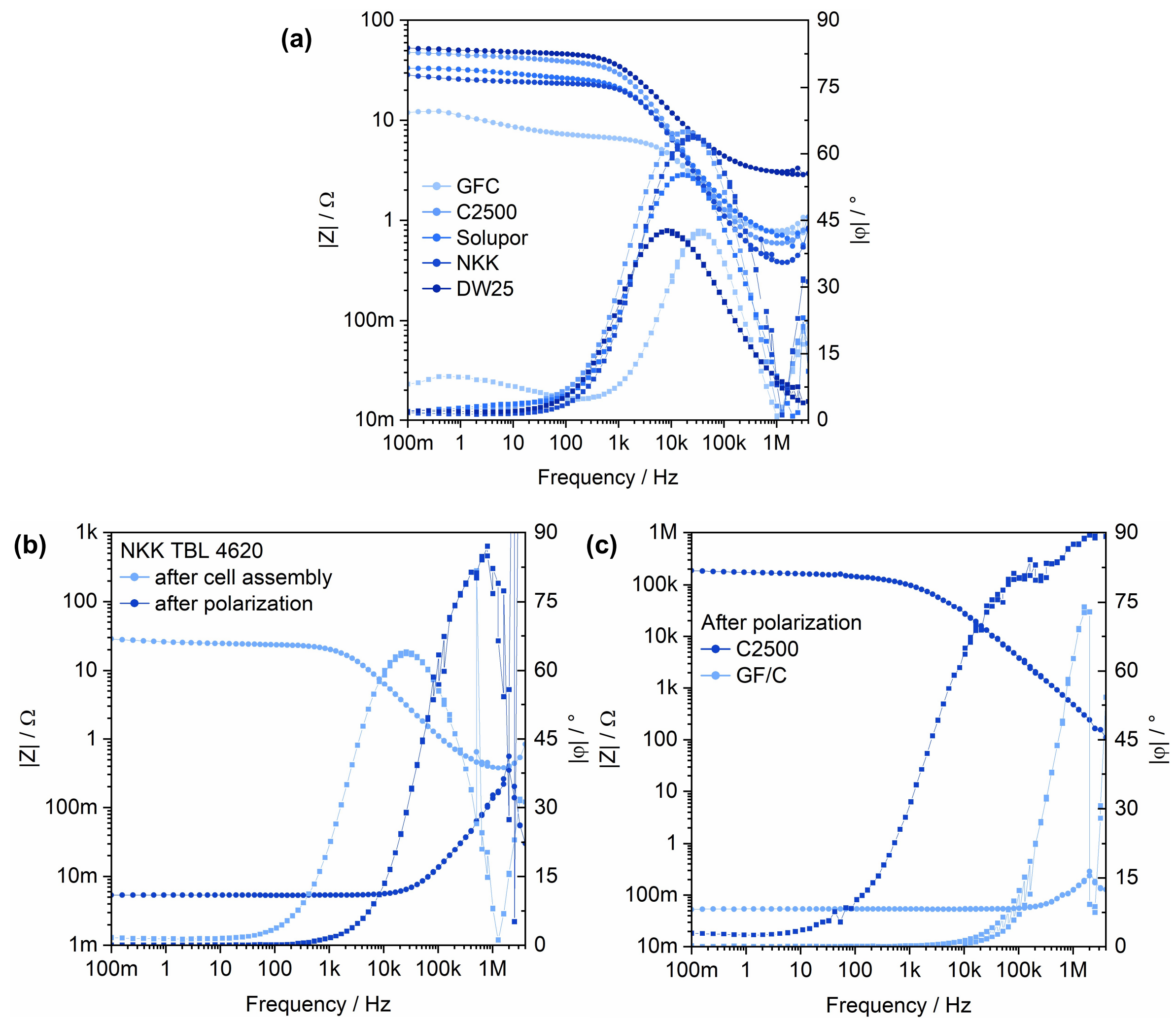

Figure S11: Impedance spectra of Li|Li cells comprising different separators (a) after cell assembly, (b) NKK separator and (c) Celgard 2500 and GF/C after cell assembly and polarization at 1 mA cm^{-2} (short circuit in case of GF/C and NKK).

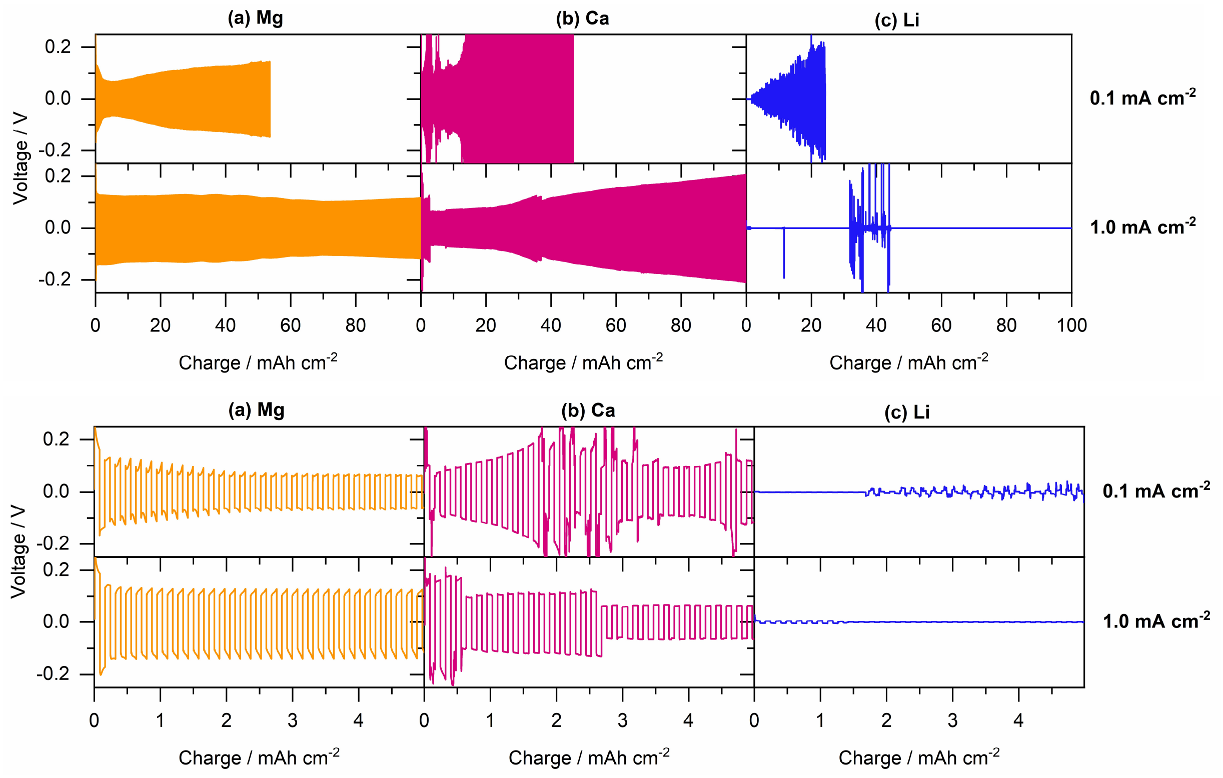

Figure S12: Polarization of Mg|Mg, Ca|Ca and Li|Li cells at 0.1 and 1 mA cm^{-2} applying a GF/C separator (150 µl electrolyte). Bottom: Zoom.

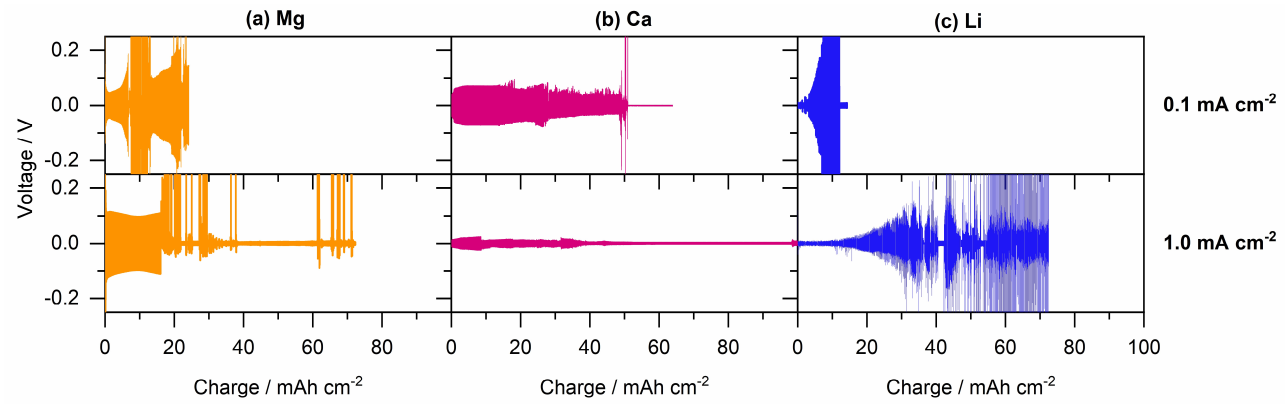

Figure S13: Zoom-out of Figure 7. Polarization of Mg|Mg, Ca|Ca and Li|Li cells at 0.1 and 1 mA cm^{-2} applying a Celgard 2500 separator (50 µl electrolyte).

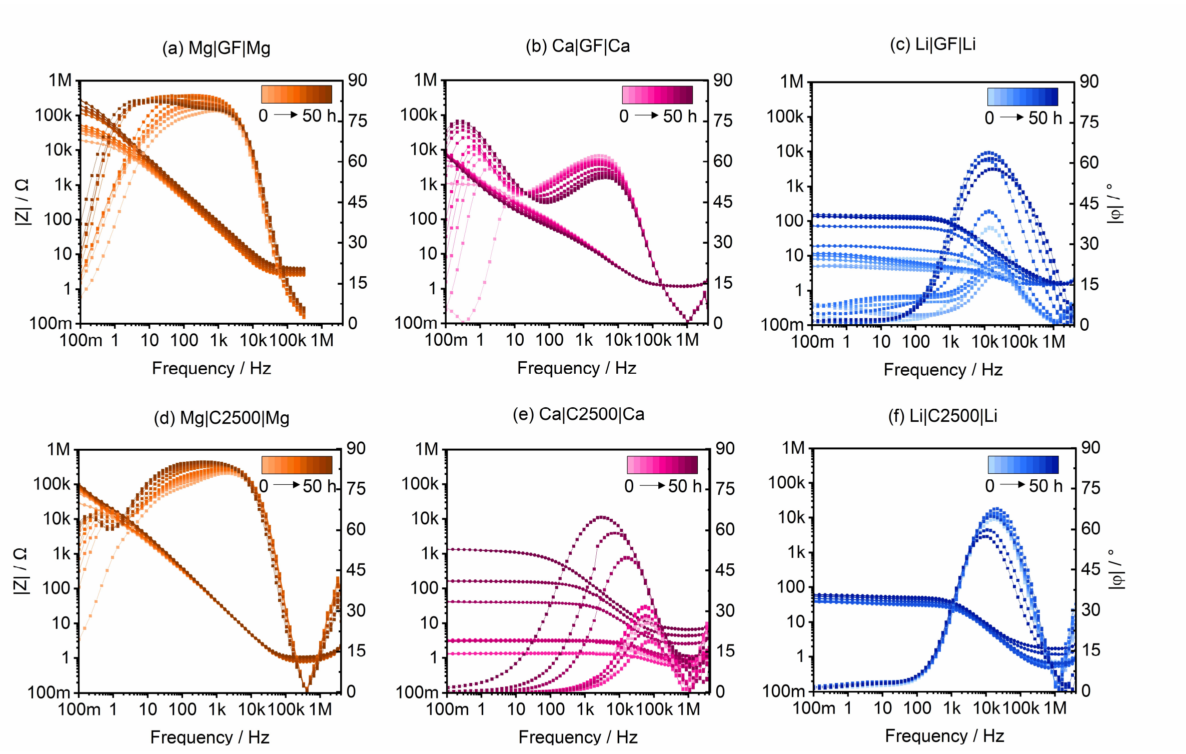

Figure S14: Impedance spectra evolution during 50 h OCV in Mg|Mg, Ca|Ca and Li|Li cells with (a-c) two layers GF (250 µl) and (d-f) one layer C2500 separator (50 µl), respectively.

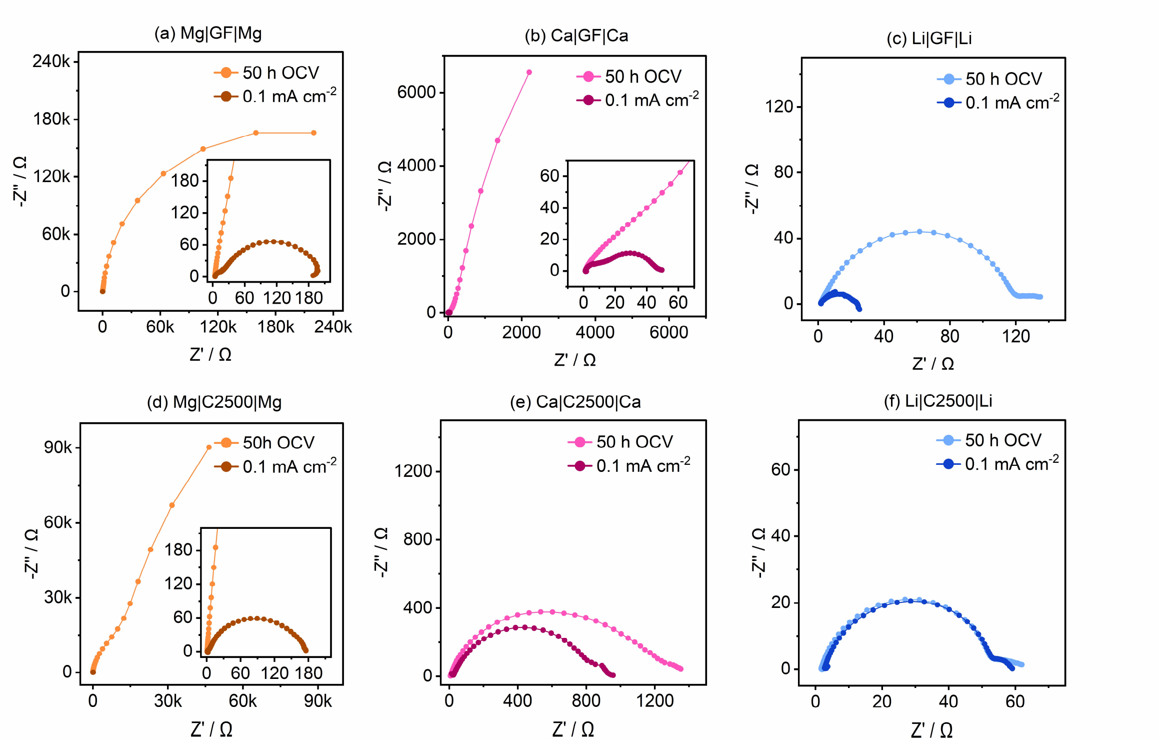

Figure S15: Corresponding Bode plots to Fig. 9: Comparison of the final impedance spectra after 50 h OCV and the first impedance spectra during polarization with 0.1 mA cm^{-2} in Mg|Mg, Ca|Ca and Li|Li symmetrical cells with two layers of Whatman (a-c, 250 µl) and one layer of Celgard (d-e, 50 µl), respectively.

Adsorption layer formation during OCV (GF/C separator)

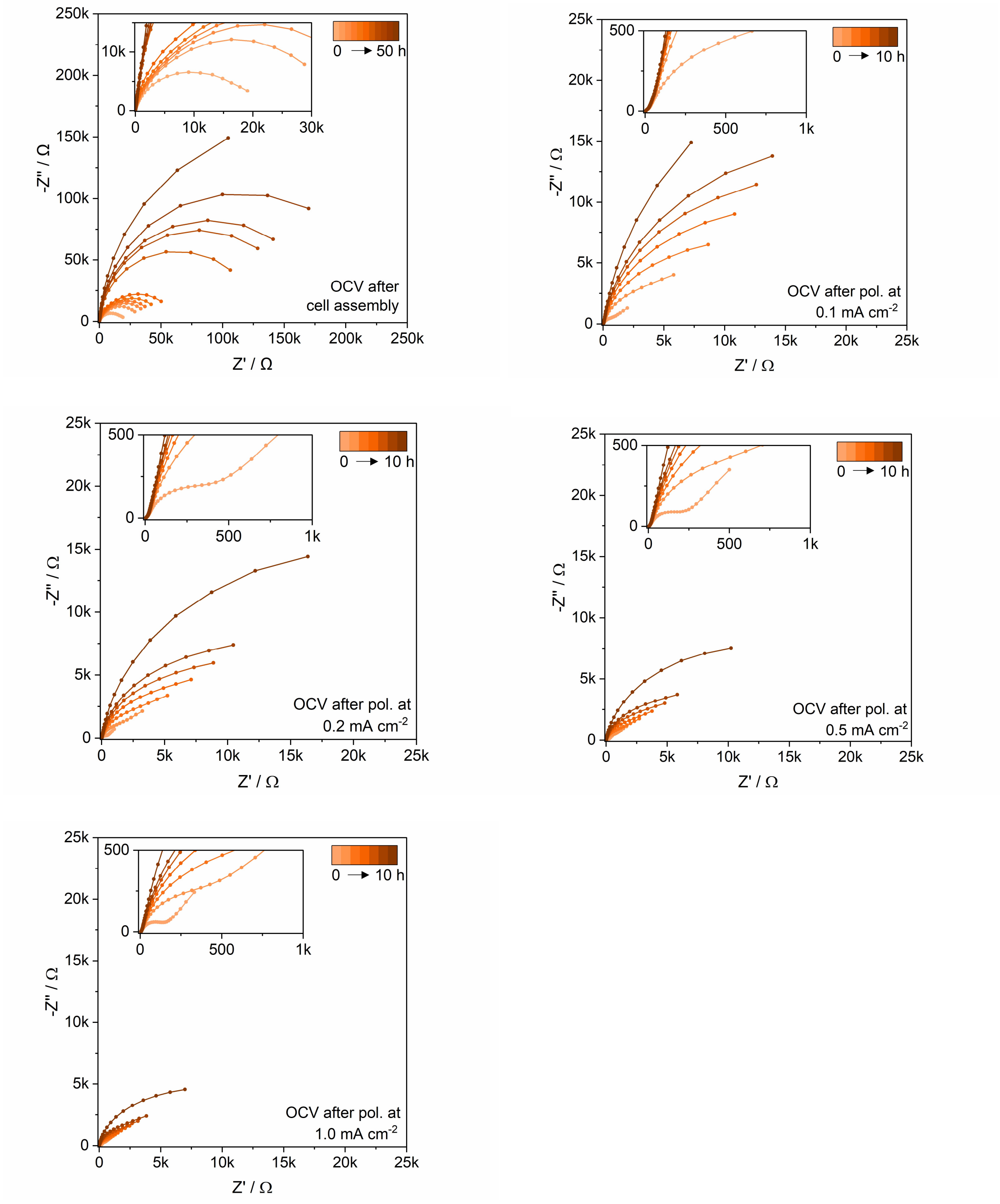

Figure S16: Impedance spectra of a Mg|Mg cell (2x GF/C, 200 µl) during OCV (50 h initial, 10 h intermittent during polarization).

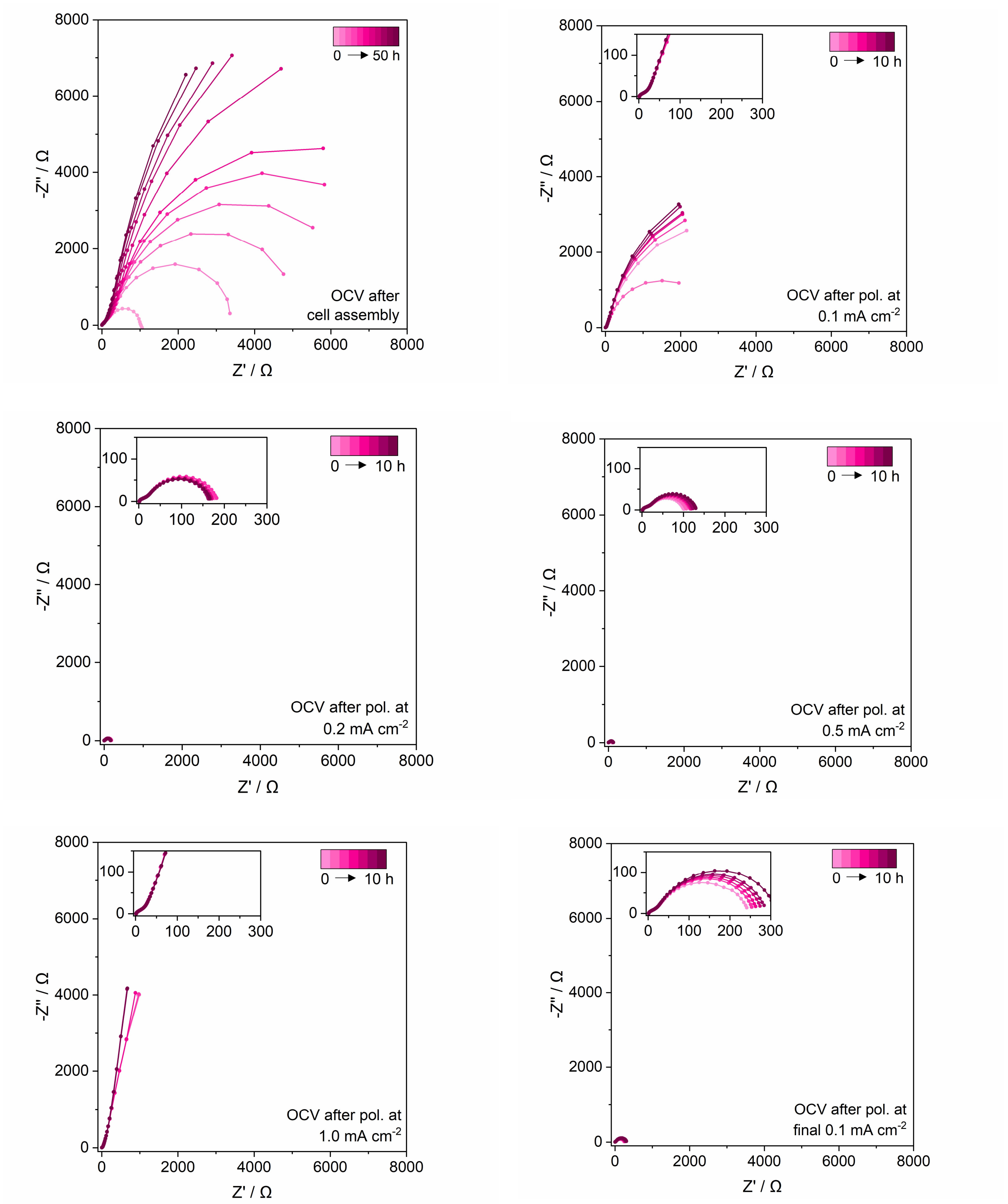

Figure S17: Impedance spectra of a Ca|Ca cell (2x GF/C, 200 µl) during OCV (50 h initial, 10 h intermittent during polarization). While adding to a huge contribution initially, the adsorption layer hardly forms after surface layer formation.

Adsorption layer formation during OCV (C2500 separator)

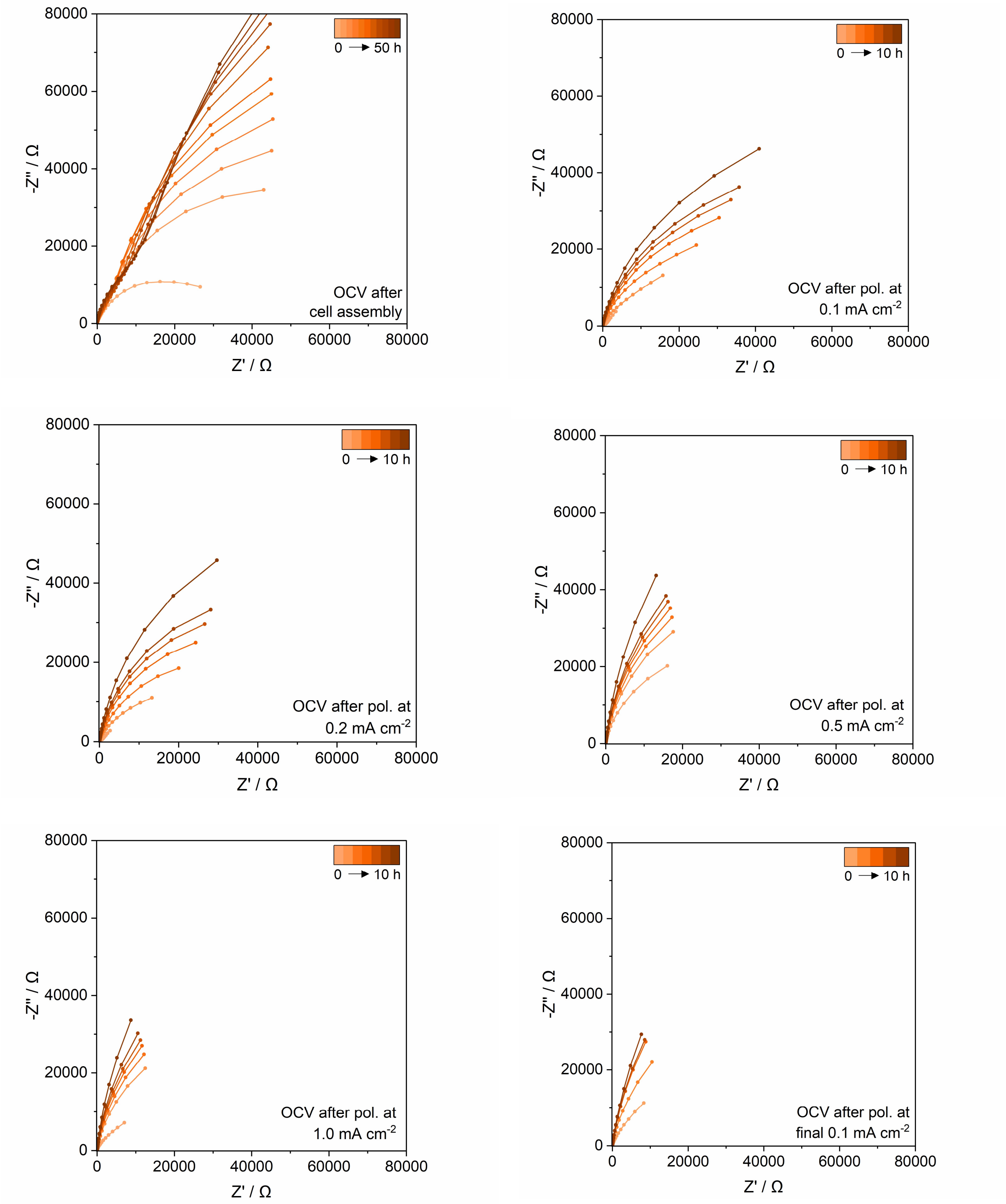

Figure S18: Impedance spectra of a Mg|Mg cell (C2500, 50 µl) during OCV (50 h initial, 10 h intermittent during polarization).

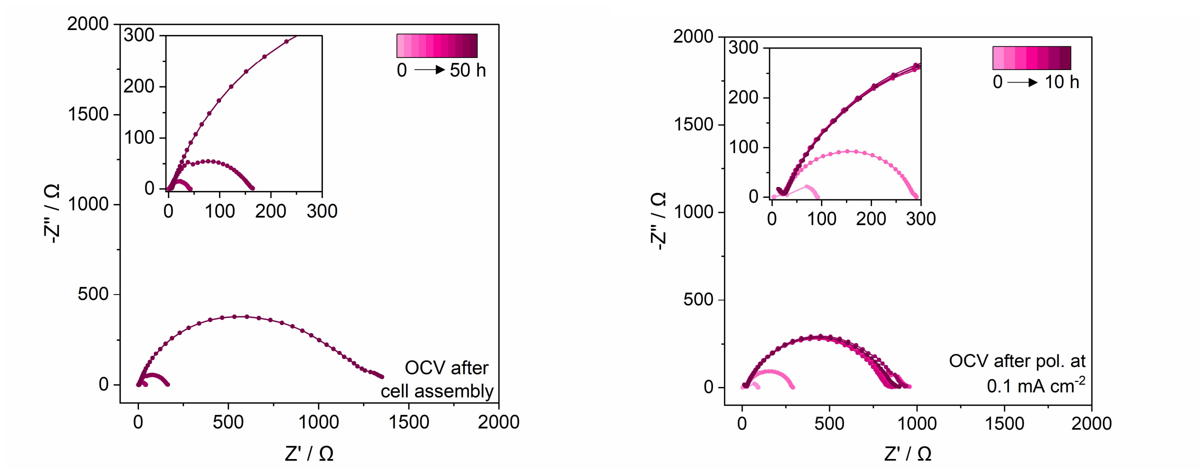

Figure S19: Impedance spectra of a Ca|Ca cell (C2500, 50 µl) during OCV (50 h initial, 10 h intermittent during polarization).

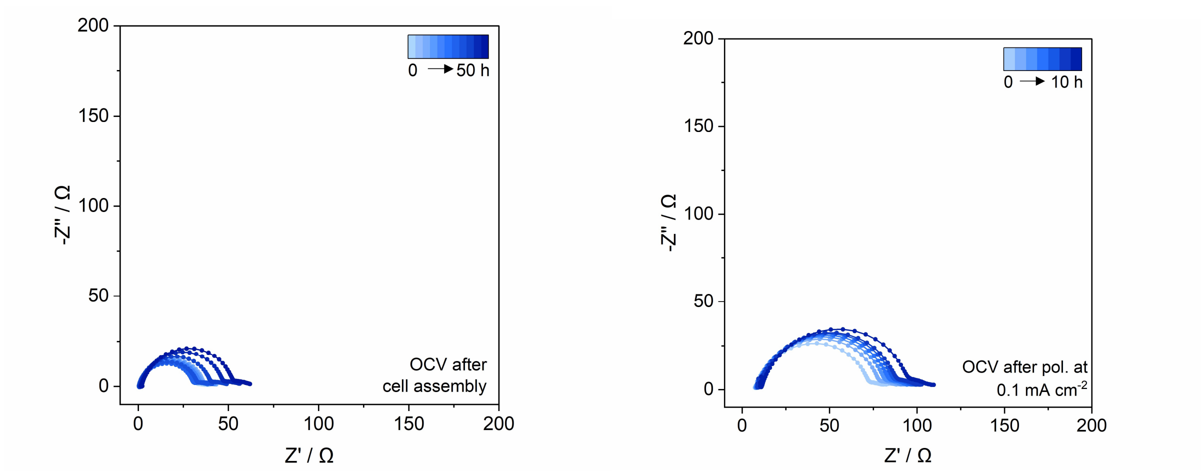

Figure S20: Impedance spectra of a Li|Li cell (C2500, 50 µl) during OCV (50 h initial, 10 h intermittent during polarization).

EIS during polarization (Celgard 2500)

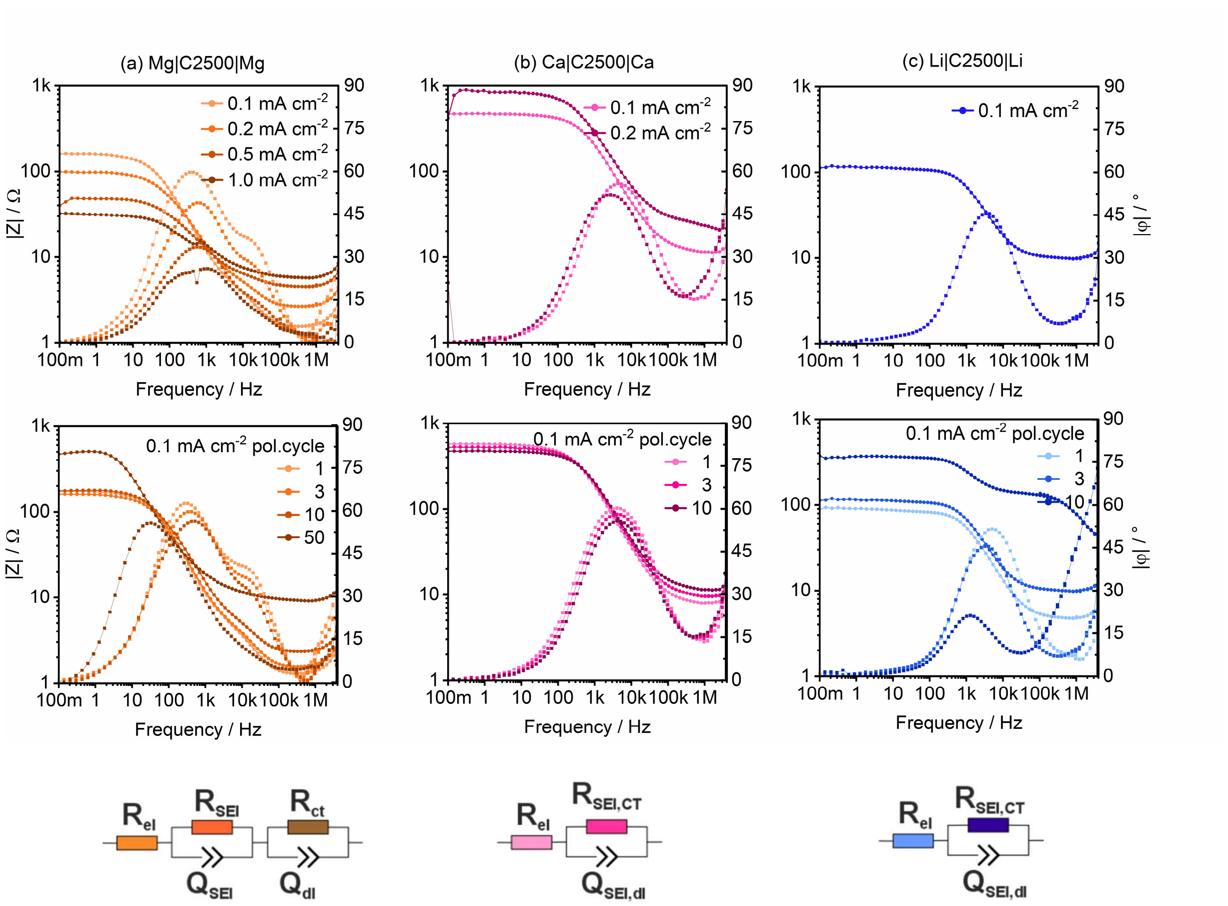

Figure S21: Impedance spectra, equivalent circuit model (ECM) and resistance evolution of (a) Mg|Mg, (b) Ca|Ca and (c) Li|Li cells during polarization at different current densities and cycle number (C2500, 50 µl).

Ca anode: Surface scraping

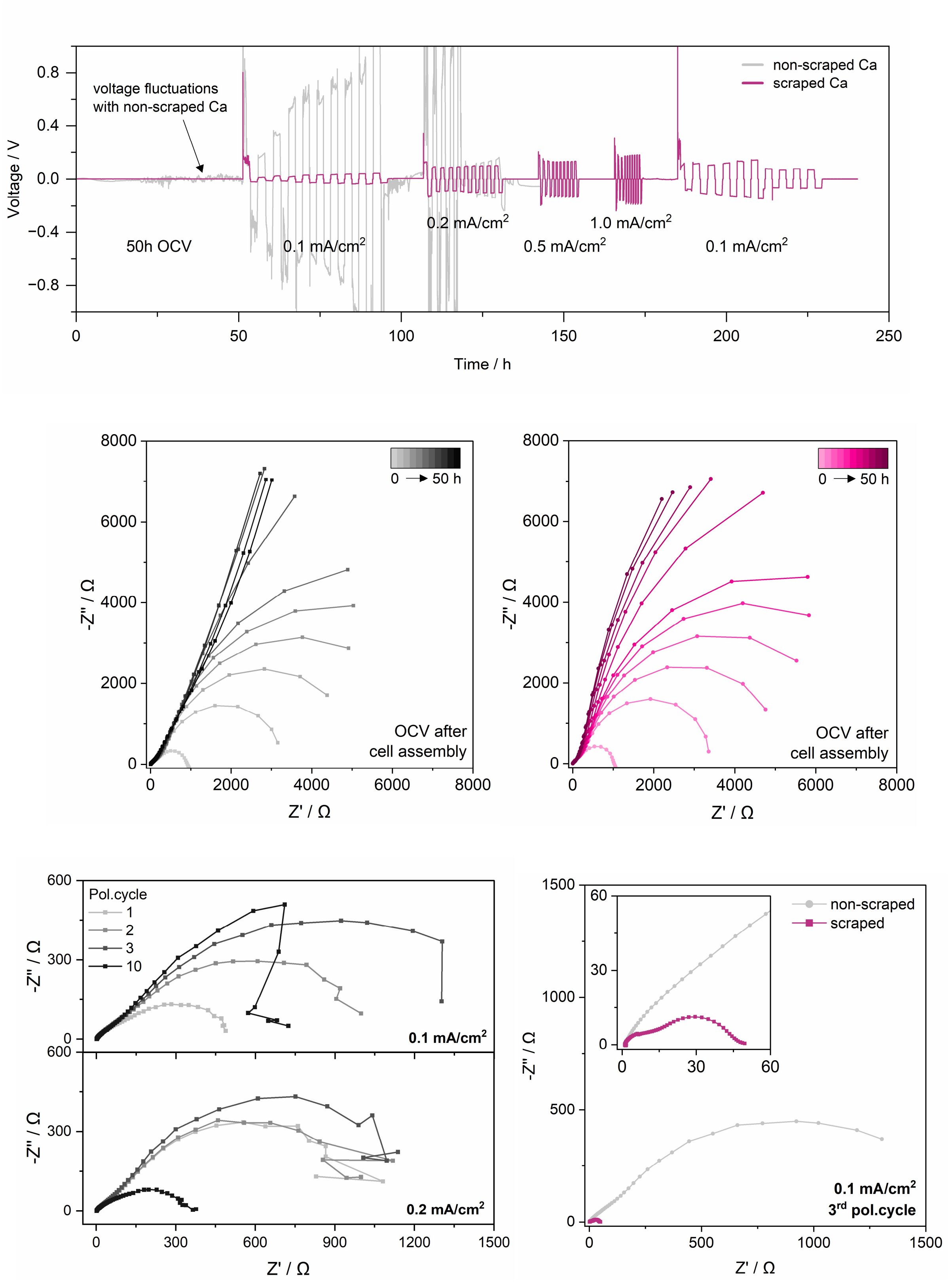

Figure S22: (a) OCV and overpotential evolution of Ca|Ca cells (2x GF/C, 250 µl) with scraped and non-scraped Ca pellets during 50 h rest and subsequent polarization.

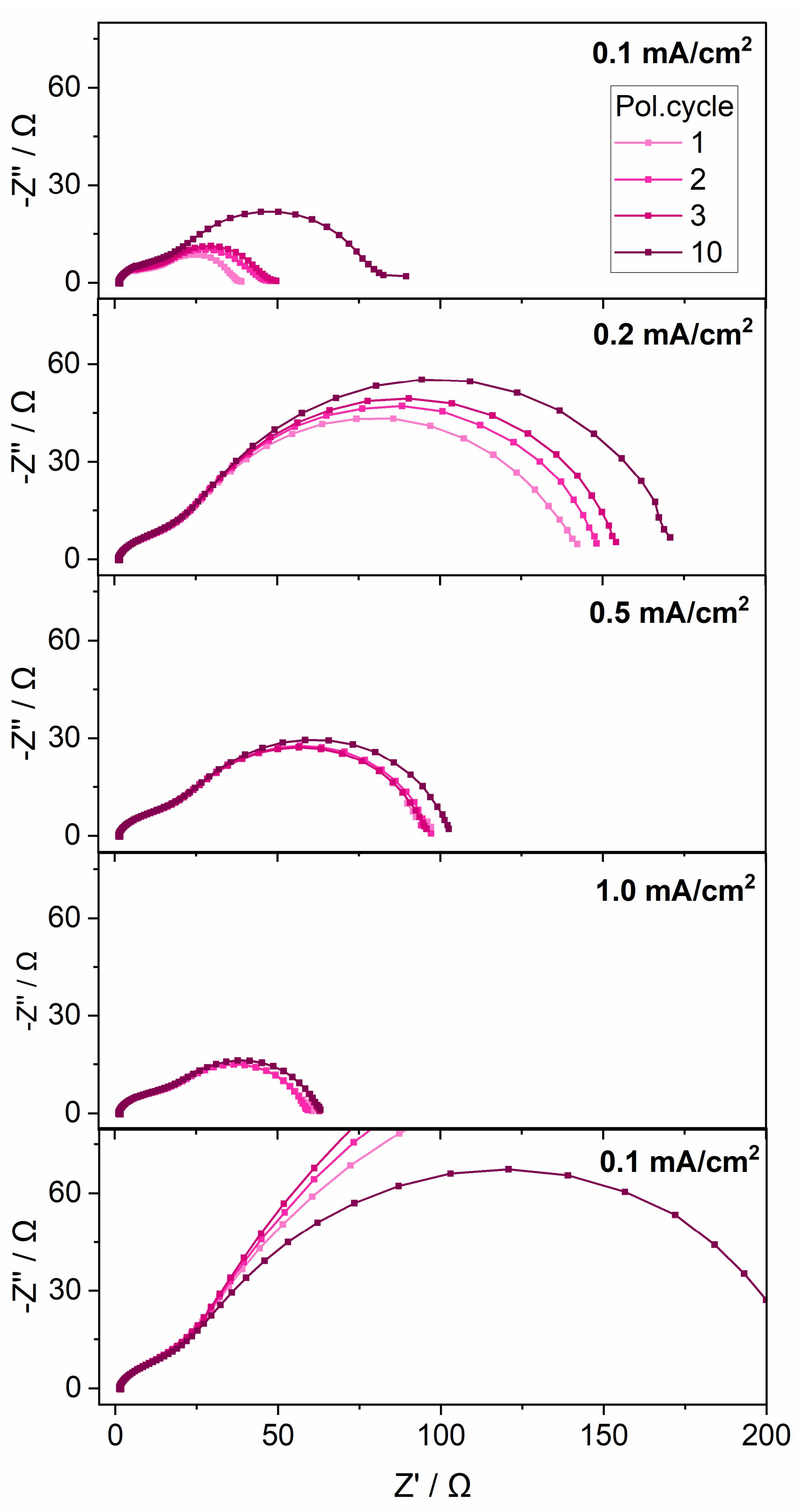

Figure S23: Nyquist spectra of a Ca|Ca cell (2x GF/C, 250 µl) with scraped Ca pellets during polarization at different current densities.

References

(1) Zhao-Karger, Z.; Bardaji, E. G.; Fuhr, O.; Fichtner, M. New class of non-corrosive, highly efficient electrolytes for rechargeable magnesium batteries. *J. Mater. Chem. A* **2017**, *5* (22), -. DOI: 10.1039/C7TA02237A.
(2) Li, Z.; Fuhr, O.; Fichtner, M.; Zhao-Karger, Z. Towards stable and efficient electrolytes for room-temperature rechargeable calcium batteries. *Energy & Environmental Science* **2019**, *12* (12), 3496-3501. DOI: 10.1039/c9ee01699f.
(3) Tang, K.; Du, A.; Dong, S.; Cui, Z.; Liu, X.; Lu, C.; Zhao, J.; Zhou, X.; Cui, G. A Stable Solid Electrolyte Interphase for Magnesium Metal Anode Evolved from a Bulky Anion Lithium Salt. *Adv Mater* **2020**, *32* (6), e1904987. DOI: 10.1002/adma.201904987.
